# Atorvastatin improves spermatogenesis in murine and in vitro human chronic orchitis models through restoring blood-testis barriers

**DOI:** 10.1038/s41420-025-02749-6

**Published:** 2025-11-06

**Authors:** Linzi Ma, Jiyu Chen, Qi Li, Yongtong Zhu, Shaofang Ren, Ke Song, Zhaokai Yao, Xinyan Yang, Yi Zheng, Zhaoting Liu, Zehui Wang, Kai Miao, Shiling Chen, Xiao-Yang Zhao, Fang Luo

**Affiliations:** 1 Center for Reproductive Medicine, Department of Gynecology and Obstetrics, Nanfang Hospital, Southern Medical University, Guangzhou, China; 2 State Key Laboratory of Organ Failure Research, Department of Developmental Biology, School of Basic Medical Sciences, Southern Medical University, Guangzhou, China; 3 Guangdong Second Provincial General Hospital, Postdoctoral Research Station of Basic Medicine, School of Medicine, Jinan University, Guangzhou, Guangdong China; 4 Department of Obstetrics and Gynecology, Guangdong Provincial Key Laboratory of Major Obstetric Diseases, Guangdong Provincial Clinical Research Center for Obstetrics and Gynecology, Guangdong-Hong Kong-Macao Greater Bay Area Higher Education Joint Laboratory of Maternal-Fetal Medicine, The Third Affiliated Hospital, Guangzhou Medical University, Guangzhou, Guangdong China; 5 Reproductive Medicine Center, Shunde Hospital of Southern Medical University, Shunde, China; 6 Centre for Precision Medicine Research and Training, Faculty of Health Sciences, University of Macau, Macau, China; 7 State Key Laboratory of Organ Failure Research, School of Laboratory Medicine and Biotechnology, Southern Medical University, Guangzhou, Guangdong China; 8 Guangdong Provincial Key Laboratory of Construction and Detection in Tissue Engineering, Southern Medical University, Guangzhou, China; 9 Key Laboratory of Mental Health of the Ministry of Education, Guangzhou, China; 10 Guangdong-Hong Kong Joint Laboratory for Psychiatric Disorders, Guangzhou, China; 11 Department of Gynecology, Zhujiang Hospital, Southern Medical University, Guangzhou, Guangdong China; 12 National Clinical Research Center for Kidney Disease, Guangzhou, China

**Keywords:** Phenotypic screening, Tight junctions

## Abstract

Chronic orchitis, which stems from autoimmune responses and infections, significantly impairs the testicular niche and affects male fertility. However, there is a lack of effective therapeutic drugs. In this study, we aimed to explore whether atorvastatin can be used for the treatment of chronic orchitis and the underlying mechanism based on our previous findings and detection using the database. We utilized mouse models of chronic orchitis induced by both autoimmune and infectious causes to demonstrate that atorvastatin can markedly improve spermatogenesis and fertility. Mechanism studies through single-cell transcriptomic analysis, target gene knockdown, quantitative proteomics, and small molecule interference revealed that atorvastatin suppressed the Rac1/AP1/MMPs pathway via inhibition of 3-hydroxy-3-methylglutaryl coenzyme A reductase (HMGCR) in Sertoli cells, thereby restoring the blood-testis barrier (BTB) and spermatogenesis. More importantly, atorvastatin also re-established the expression of BTB-associated proteins and increased the germ cell numbers in cultured human testis tissues. Collectively, our study reveals the essential impact of atorvastatin in improving spermatogenesis in murine and human chronic orchitis models through restoring BTB and suggests that atorvastatin as a promising agent for the clinical treatment of chronic orchitis.

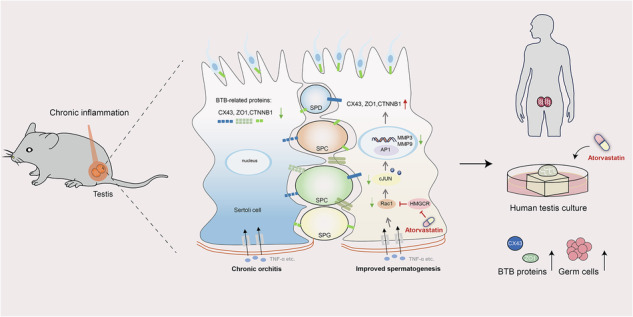

## Introduction

The prevalence of infertility keeps increasing worldwide. According to a newly released report by the World Health Organization, 17.5% of the adult population would encounter infertility during their lifetime [1]. Among them, male factor accounts for about 50% of the sterility, representing a global public health concern that demands attention and action [2].

Infections and inflammations of the male reproductive system are recognized as important etiological factors of infertility. Previous reports indicate that infections of the reproductive system account for 6 to 15% of male infertility cases, and even surge to 30% in regions with inadequate healthcare [3, 4]. In particular, orchitis can directly disrupt spermatogenesis and lead to permanent decrease in both sperm quantity and quality [5]. Although traditional treatments for acute orchitis can eradicate pathogenic microbes, the impaired spermatogenesis caused by inflammation is irreversible. Epidemiological studies show that 22–37% of patients with acute epididymo-orchitis developed into oligozoospermia, while 6–8% exhibit severe azoospermia [6, 7]. For patients with chronic orchitis, there are no standard clinical treatment guidelines. Several attempts have been made to treat chronic testicular inflammatory lesions and restore reproductive function, including glucocorticoids, nonsteroidal anti-inflammatory drugs (NSAIDs), and mast cell stabilisers. However, these interventions have yielded unsatisfactory results, often accompanied by significant side effects after prolonged use, which limits their translational value [5, 8, 9]. Therefore, there is an urgent need for effective therapeutic interventions for treating chronic orchitis.

The mammalian testis is a typical immune-privileged organ, in which the immune response is suppressed, thereby safeguarding the immunogenic germ cells from systemic immune attack under normal physiological state. Specifically, the blood-testis barrier (BTB), which is constituted by Sertoli cells, insulates the germ cells in the seminiferous tubules from the immune environment and thus provides the structural foundation for the immune privilege [10]. Furthermore, Sertoli cells and Leydig cells play immunosuppressive roles by secreting immune regulatory factors, which are crucial for preservation normal spermatogenesis [11, 12]. However, the onset of inflammation can disrupt the testicular immune homeostasis with the pro-inflammatory factors, leading to an impaired spermatogenesis microenvironment, including disrupted BTB and reduced gonadal secretion [13]. Hence, developing strategies to effectively enhance spermatogenesis along with ameliorating inflammation might be the crucial for chronic orchitis therapy.

Statins, 3-hydroxy-3-methylglutaryl coenzyme A reductase (HMGCR) inhibitors, were originally discovered as drugs for the treatment of hypercholesterolemia [14]. Prior studies have also reported that they play an important role in angiogenesis, cell proliferation, as well as inflammation and immune regulation by mediating the prenylation of small GTPase family members such as Ras, Rac, Rab, and Rho, which are involved in intracellular signal transduction and the regulation of transcription factor activity. This is achieved by preventing the synthesis of downstream lipid isoprenoid intermediates, including farnesyl pyrophosphate and geranylgeranyl pyrophosphate [15–17]. Previous studies of statins in the reproductive system have shown that Rosuvastatin and Simvastatin can exert anti-inflammatory and antioxidant effects and attenuate testicular damage in diabetic and cadmium toxicity rat models [18, 19]. Moreover, our previous study demonstrated that statins promoted the self-renewal of spermatogonial stem cells (SSCs) and inhibited their inflammation and apoptosis [20], which suggests their potential in treating orchitis. Atorvastatin, as an optimized third-generation statin, has a strong lipid-lowering effect with more time selectivity and no nephrotoxicity, which made it be the most commonly used statin in clinical practices, showing better probability of clinical translation.

In the present study, using two chronic orchitis mouse models, we demonstrated that atorvastatin administration could alleviate inflammation levels while remarkably enhancing impaired spermatogenesis. Mechanistically, we found that atorvastatin might restore the BTB integrity through mevalonate cascades and Rac1/AP1/MMPs axis. Notably, atorvastatin also re-established the BTB and increased germ cell numbers in cultured human testis tissues. In conclusion, our findings suggested that atorvastatin might represent a novel therapeutic approach for chronic orchitis, holding significant potential for clinical translation.

## Results

### Atorvastatin restores the impaired spermatogenesis in EAO mice

**Fig. 1:**
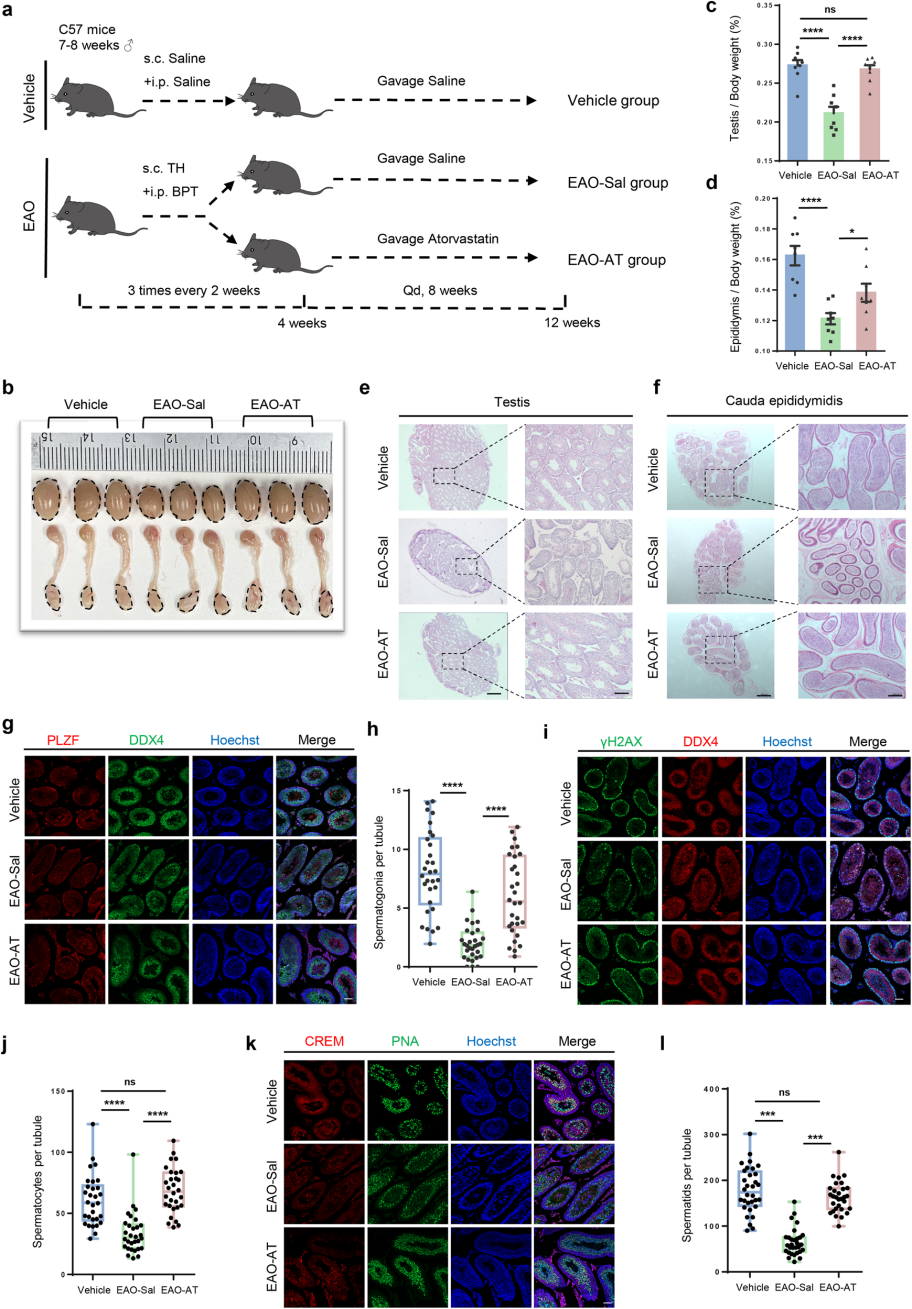
Atorvastatin treatment improves spermatogenesis in EAO mice. **a** Schematic illustration of the experimental design for the gavage administration of atorvastatin in EAO mice. S.c. subcutaneous injection, i.p. intraperitoneal injection, TH testicular homogenate, BPT Bordetella pertussis toxin. **b** Bright field images of mouse testis and epididymis in Vehicle, EAO-Sal and EAO-AT groups. **c**, **d** Coefficient of testis (testis/body weight) and epididymis (epididymis/body weight) in Vehicle, EAO-Sal and EAO-AT groups, *n* = 8. Histological sections of testis (**e**) and cauda epididymidis (**f**) in Vehicle, EAO-Sal and EAO-AT groups, stained with hematoxylin and eosin (H&E), dotted box represents the enlarged area shown. Scale bar, 500 μm (left), 200 μm (right). Representative immunofluorescence images (**g**, **i**, **k**) and quantitative analysis (**h**, **j**, **l**) of the number of spermatogonia, spermatocytes, and spermatids in the tubule of testis from 30 randomly selected fields. DDX4 labeled germ cells, DDX4 and PLZF co-staining labeled spermatogonia, DDX4 and γH2AX co-staining labeled spermatocytes, CREM, and PNA co-staining labeled spermatids. Scale bar, 50 μm, *n* = 5 testis samples per group. Data are presented as mean ± SEM, *P < 0.05, ****P < 0.0001, ns indicates no statistical significance.

To explore the therapeutic approach for chronic orchitis, we initially established a classical model of experimental autoimmune orchitis [21] (EAO), and then assessed whether atorvastatin administration could restore spermatogenesis in EAO mice (Fig. 1a).

Atorvastatin was administered in disease models with different dosages vary from 1 to 10 mg/kg [18, 19, 22], therefore, we first determined the optimal concentration of atorvastatin. EAO mice treated with different concentrations of atorvastatin (EAO-AT 1/5/10) showed dose-dependent treatment effects compared to the EAO mice treated with Saline (EAO-Sal), including changes in the size and coefficient of testis and epididymis, among which 10 mg/kg was the most effective one, as mice from this group almost recovered to the same level as the Vehicle group (treated with Saline in all the administration) (Fig. S1a–c). Remarkably, the vacuolation observed in the seminiferous epithelium, accompanied by a loss of germ cells in the EAO-Sal group, was mitigated following intervention with varying doses of atorvastatin (Fig. S1d). Likewise, a dose-dependent increase in sperm density was evident in the cauda epididymidis (Fig. S1e). Hepatotoxicity and nephrotoxicity analysis demonstrated that there were no obvious structural abnormalities even with the highest dosage of atorvastatin, i.e., 10 mg/kg, suggesting that atorvastatin may not have potential side effects at therapeutic dosages (Fig. S1f–j). Therefore, 10 mg/kg of atorvastatin was selected for all the subsequent mouse experiments.

It was found that atorvastatin partially restored the size and coefficients of the testis and epididymis in chronic orchitis mice (Fig. 1b–d), repaired the damage histopathologically, and increased the number of testicular germ cells (Fig. 1e, f). We further evaluated various spermatogenic cell types, including DDX4^+^ germ cells, PLZF^+^ spermatogonia (Fig. 1g, h), γH2AX^+^ spermatocytes (Fig. 1i, j), and CREM^+^PNA^+^ spermatids (Fig. 1k, l). Notably, it was shown that the number of all these spermatogenic cells was significantly increased in the EAO-AT group compared to the EAO-Sal group (spermatogonia: EAO-Sal=2.02 ± 0.27, EAO-AT = 6.05 ± 0.61; spermatocytes: EAO-Sal = 33.74 ± 3.06, EAO-AT = 68.70 ± 3.38; spermatids: EAO-Sal = 64.95 ± 5.49, EAO-AT = 163.60 ± 6.15). More importantly, the number of spermatocytes and spermatids was comparable to those in the Vehicle group, suggesting that atorvastatin was beneficial for improving the impaired spermatogenesis in EAO mice.

### Atorvastatin elevates the sperm quality of EAO mice and restores their fertility

**Fig. 2:**
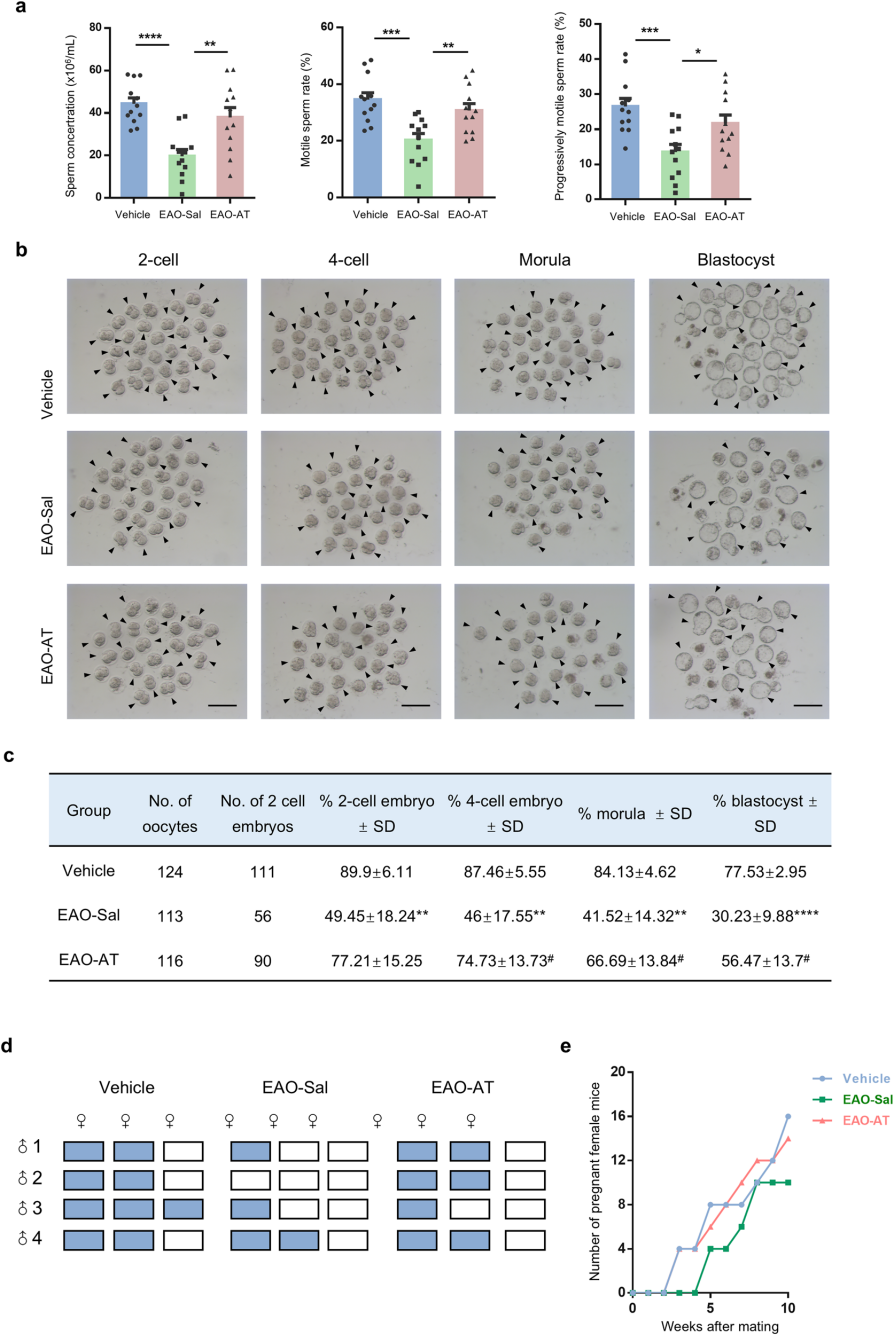
Atorvastatin improves semen parameters and partially restores the fertility of EAO mice. **a** Sperm concentration and sperm motility in Vehicle, EAO-Sal and EAO-AT groups, *n* = 12. **b** Bright field diagram of 2-cell, 4-cell, morula, and blastocyst derived from sperm in Vehicle, EAO-Sal and EAO-AT groups by in vitro fertilization (IVF). Arrows indicate normal developing embryos. Scale bar, 200 μm. **c** The trilinear table shows all the embryo preimplantation development data of IVF in Vehicle, EAO-Sal and EAO-AT groups, * indicates Vehicle vs EAO-Sal group and # indicates EAO-Sal vs EAO-AT group, *n* = 4. **d** Presence of vaginal plugs (blue boxes) in female mice at childbearing age after timed mating with male mice from Vehicle, EAO-Sal and EAO-AT groups. **e** Number of pregnant female mice when natural mating with male mice from Vehicle, EAO-Sal and EAO-AT groups for 10 weeks, *n* = 4. Data are presented as mean ± SEM, */^#^*P* < 0.05, ** *P* < 0.01, ***P < 0.001, ****P < 0.0001.

To evaluate whether atorvastatin treatment could improve the quality of spermatozoon, computer-assisted sperm parameter assays and in vitro fertilization were conducted. The results of sperm parameter assays showed that atorvastatin significantly enhanced sperm concentration (Vehicle = 44.38 ± 2.77, EAO-Sal = 19.67 ± 3.13, EAO-AT = 37.91 ± 4.65) and sperm motility in EAO mice, with a notable increase in the progressively motile sperm rate (Vehicle = 26.49 ± 2.30, EAO-Sal = 13.49 ± 2.18, EAO-AT = 21.58 ± 2.47) (Fig. 2a). Consistently, atorvastatin intervention effectively ameliorated the fertilization potential of sperm with impaired quality from EAO mice, significantly enhanced embryo development rates at all the stages. Specifically, the 2-cell rate increased from 49.45 ± 18.24% to 77.21 ± 15.25%, the 4-cell rate from 46 ± 17.55% to 74.73 ± 13.73%, the morula rate from 41.52 ± 14.32% to 66.69 ± 13.84%, and the blastocyst rate from 30.23 ± 9.88% to 56.47 ± 13.7% (Fig. 2b, c).

In addition, different groups of male mice were paired with normal fertile female mice to test the fertility through natural mating. Compared to the EAO-Sal group, atorvastatin treatment resulted in an increased occurrence of vaginal plug and pregnancies (Fig. 2d, e), as well as more litter births within the same mating period (Fig. S2a), suggesting that atorvastatin effectively enhanced the fertility of EAO mice. Subsequently, we monitored the body weight of the offspring from each group for 1 month with weighing them once a week. The results showed that no significant difference was observed in either male or female mice (Fig. S2b), suggesting that atorvastatin treatment not only restored the fertility in EAO mice, but also did not cause abnormal growth in the offspring. Additionally, during the natural mating process, no drug intervention was administered. Approximately 3 months after natural mating, samples were collected to assess testicular and epididymal coefficients, along with sperm parameter assays. Notably, the coefficients of the testis and epididymis in the EAO-AT group were still higher than those in the EAO-Sal group (Fig. S2c). Additionally, the sperm concentration and motility in the EAO-AT group were also significantly elevated than those in the EAO-Sal group (Fig. S2d–f), implying that the effect of atorvastatin was not transient but could persist for 3 months after withdrawal. Furthermore, atorvastatin treatment enhanced the serum testosterone level to a certain extent (Fig. S2g). Collectively, these findings suggest that atorvastatin treatment could improve sperm quality and enhance the fertility of EAO mice.

### Atorvastatin ameliorated testicular inflammation

**Fig. 3:**
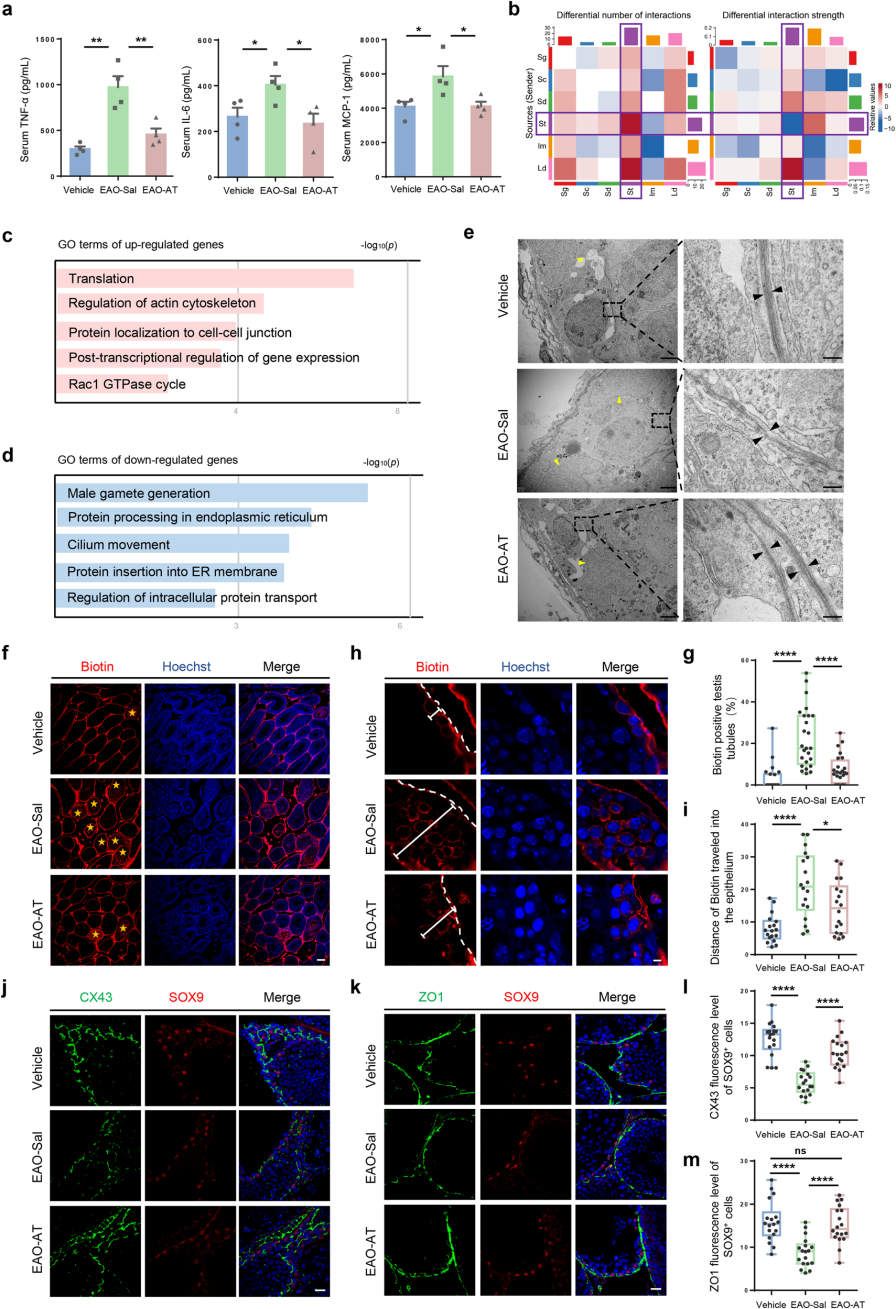
Atorvastatin ameliorated testicular inflammation and restored damaged BTB in EAO mice. **a** TNF-α, IL-6, and MCP-1 levels in serum of mice from Vehicle, EAO-Sal and EAO-AT groups measured by ELISA, *n* = 4. **b** Heatmap of differential interaction number (left) and strength (right) between EAO mice and normal control group. In the color bar, red (or blue) represents increased (or decreased) signaling in the EAO compared to the normal control group. **c**, **d** Enriched GO terms in the upregulated and downregulated genes of Sertoli cells from EAO mice versus with normal control. **e** Representative TEM images of testis from Vehicle, EAO-Sal and EAO-AT groups, showing BTB ultrastructure. Black arrows indicate tight junctions, while yellow arrows indicate Sertoli cell nuclei. Bars: left 2 µm; right 250 nm. **f**, **g** Immunofluorescence of biotin (red) and biotin-positive seminiferous tubules percentage in testis of Vehicle, EAO-Sal and EAO-AT groups from 24 randomly selected fields. Yellow asterisks indicate biotin-positive seminiferous tubules. Scale bar, 50 μm. *n* = 4 testis samples per group. **h**, **i** Immunofluorescence staining image and statistical histogram of the distance of biotin traveled into tubules in each group from 18 seminiferous tubules. The dashed lines indicate the boundary of the seminiferous tubules, and the solid lines indicate the biotin running distance. Scale bar, 5 μm. *n* = 4 testis samples per group. **j**, **k** Immunofluorescence of CX43 and ZO1 (green) co-stained with SOX9 (red) in Vehicle, EAO-Sal and EAO-AT groups. SOX9 labeled Sertoli cells, CX43 labeled Connexin43, ZO1 labeled Tight junction protein 1. Scale bar, 5 μm. **l**, **m** Quantitative analysis for immunofluorescence intensity of CX43 and ZO1 from 18 randomly selected fields. *n* = 4 testis samples per group. Data are presented as mean ± SEM, *P < 0.05, **P < 0.01, ****P < 0.0001, ns indicates no statistical significance.

Chronic inflammation, accompanied by a marked elevation of inflammatory factors such as TNF-α, IL-6, and MCP-1, disrupts the spermatogenic niche and thereby impairs sperm maturation and viability [4]. Therefore, we first assessed whether atorvastatin could reduce inflammation levels. ELISA analysis showed that the serum inflammatory factors were notably reduced in EAO mice after atorvastatin treatment (Fig. 3a). Consistently, mRNA expression levels of inflammatory factors in the testis demonstrated similar results (Fig. S3a). Furthermore, we examined the F4/80^+^ macrophages and found a marked decrease after atorvastatin intervention (Fig. S3b, c). These results suggested that atorvastatin alleviated the testicular inflammation in EAO mice.

### Atorvastatin restored the BTB integrity in EAO mice

To study how atorvastatin optimized the spermatogenic microenvironment in the EAO mice, we first analyzed the testicular niche in EAO mice using single-cell RNA-seq data from the previous report to find clues [23]. After cell identity annotation and distribution analysis (Fig. S3d–f), we estimated the changed cell communications between control and EAO group by CellChat analysis. The results showed that Sertoli cells were the most active cells both in terms of the differential number of interactions and the differential interactions strength in both the heat map (Fig. 3b) and the chordal plot (Fig. S3g). Furthermore, GO analysis of Sertoli cells in EAO mice showed that the up-regulated genes were mainly enriched in pathways such as “regulation of the actin cytoskeleton” and “protein localization to cell–cell junction” (Fig. 3c), whereas down-regulated pathways such as “male gamete generation” were identified (Fig. 3d). Consistently, the expression of BTB-associated genes such as *Tjp1*, *Gja1*, *Ocln*, and *Ctnnb1* was down-regulated in Sertoli cells of EAO mice (Fig. S3h). Likewise, the disruption of BTB was observed by in vivo biotin tracing assay (Fig. S3I, j). These results indicated that dysfunctional alterations in Sertoli cells, including impaired BTB integrity, might be a leading factor contributing to infertility.

As the sole somatic cell type in the seminiferous tubules which contacts with germ cells directly, Sertoli cells are pivotal in maintaining spermatogenic microenvironment and are critical for spermatogenesis by constituting the BTB, mediating immune privilege, and providing nutrition [24, 25]. Combined with the above results, we speculated that atorvastatin might act on Sertoli cells to optimize the testicular niche and improve spermatogenesis. Consistent with our hypothesis, transmission electron microscopy (TEM) results showed that in the EAO-Sal group, the tight junctions (TJs) were disorganized, irregularly arranged and partially fragmented, reflecting loss of junctional integrity. In contrast, the EAO-AT group showed significant restoration in these pathological changes. Specifically, the TJs between adjacent Sertoli cells were typically continuous, tightly packed, and regularly arranged, comparable to the Vehicle group (Fig. 3e). Furthermore, atorvastatin notably restored the integrity of the BTB, as evidenced by a marked reduction in the number of biotin-positive seminiferous tubules (Fig. 3f, g) and a significantly shorter distance of biotin penetration into the epithelium (Fig. 3h, i) when compared to the EAO-Sal group. Similarly, the expression of gap junction protein marker CX43 [26] and the tight junction protein marker ZO1 [27] in Sertoli cells were both significantly elevated after atorvastatin administration. Noteworthy, the immunofluorescence intensity of ZO1 was close to that of the Vehicle group (Fig. 3j–m). Altogether, atorvastatin could restore the BTB integrity, which might benefit spermatogenesis.

### Atorvastatin enhanced the impaired expression of BTB-associated proteins, potentially through modulation of mevalonate cascades

**Fig. 4:**
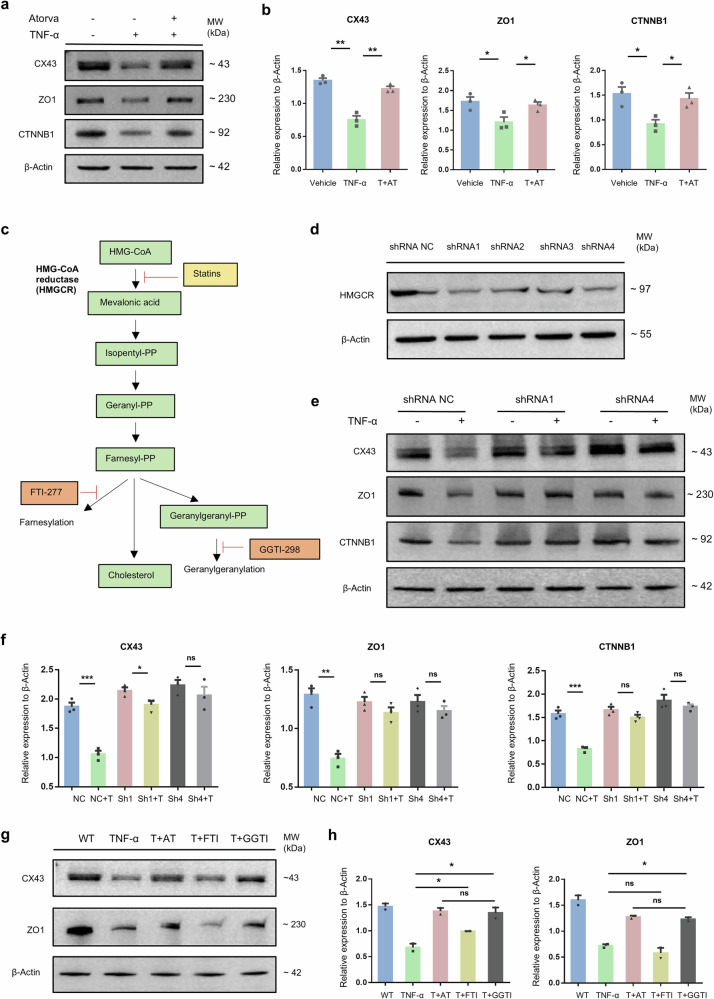
Atorvastatin functions by suppressing the HMGCR and mevalonate pathways in TM4 Sertoli cells. Representative images of western blot (**a**) and grayscale value analysis (**b**) of CX43, ZO1, and CTNNB1 in TM4 Sertoli cells (Vehicle group), and cells treated with 50 ng/mL TNF-α for 48 h (TNF-α group) or followed by 1 μM atorvastatin for 24 h (T + AT group), *n* = 3. β-Actin was used as the loading control. **c** Schematic overview of the statin target and mevalonate pathway. Main metabolites are shown in green boxes, PP means pyrophosphate. **d** Western blot analysis of HMGCR in TM4 cells after transfection with different shRNAs. **e** Representative images of western blot of CX43, ZO1 and CTNNB1 in TM4 Sertoli cells after transfection with #1 (shRNA1) or #4 shRNA (shRNA4) and being treated with 50 ng/mL TNF-α for 48 h. shRNA NC indicates non-targeting shRNA, *n* = 3. **f** Grayscale analysis in (**e**) was assessed using ImageJ. NC represents shRNA NC, NC + T represents shRNA NC treated with TNF-α, Sh1 and Sh4 represent shRNA1 and 4, whereas Sh1+T and Sh4+T represent shRNAs treated with TNF-α accordingly. **g**, **h** Representative images of western blot and grayscale value analysis of CX43 and ZO1 in TM4 Sertoli cells treated with 50 ng/mL TNF-α for 48 h (TNF-α) or followed by 1 μM atorvastatin (T + AT) or 1 μM FTI (farnesyltransferase inhibitor-277, FTI-277) or 10 μM GGTI (geranylgeranyltransferase I inhibitor-298, GGTI-298) treatment respectively (T + FTI and T + GGTI) for 24 h, *n* = 3. Data are presented as mean ± SEM, *P < 0.05, **P < 0.01, ***P < 0.001, ns indicates no statistical significance.

TNF-α plays a key role in the pathogenesis of chronic inflammation [28–30]. To facilitate the molecular mechanism study, we used TNF-α to induce mouse Sertoli cell line TM4 to mimic the inflammation of Sertoli cells in vivo [31]. Consistent with the results obtained in the mouse model, the expression levels of CX43 and ZO1, as well as another BTB-associated protein CTNNB1, were significantly downregulated in TNF-α-induced TM4 cells compared with the Vehicle group. Remarkably, atorvastatin treatment effectively restored the expression of CX43, ZO1, and CTNNB1 (Fig. 4a, b), indicating that the in vitro model could faithfully represent the therapeutic effect of atorvastatin in vivo.

It was reported that statins are the competitive inhibitors of the rate-limiting enzyme, HMG-CoA reductase (HMGCR), in the mevalonate pathway [14] (Fig. 4c). To prove the direct modulation of atorvastatin on Sertoli cells and investigate the mechanism of atorvastatin in recovering the BTB-associated proteins, we established HMGCR stable knockdown TM4 cells with four different shRNAs (Fig. S4a). As shRNA1 and shRNA4 demonstrated the best knockdown efficiency in both mRNA and protein levels, therefore, were selected for the subsequent experiments (Figs. 4d and S4b, c). The results showed that knockdown of HMGCR in TM4 cells could mimic the promoting effect of atorvastatin on the BTB-associated proteins under TNF-α-induced inflammation. In detail, upon TNF-α induction, the expression levels of CX43, ZO1, and CTNNB1 in the two knockdown groups showed no significant changes, while the shRNA-NC group exhibited a notable decrease. This suggested that atorvastatin might directly act on Sertoli cells to alleviate the inflammation-induced BTB impairment (Fig. 4e, f). Increasing evidence shows that statins could participate in a number of biological events through isoprenylation of the small G-protein family via the intermediary metabolites such as farnesyl-pyrophosphate and geranylgeranyl-pyrophosphate [15] (Fig. 4c). To further investigate whether the BTB-improvement effects of atorvastatin were related to its isoprenylation regulatory function, FTI-277 (a specific FTase inhibitor) and GGT-I298 (a specific GGTase inhibitor) were used to inhibit isoprenylation in TM4 cells (Fig. 4c). As shown in Fig. 4g, h, FTI-277 could only significantly restore the TNF-α-induced reduction in CX43, whereas GGTI-298 notably elevated the compromised expression of CX43 and ZO1 upon TNF-α-induction. Remarkably, the effect of GGTI-298 on CX43 and ZO1 protein levels was close to that of atorvastatin, suggesting that atorvastatin primarily exerts its therapeutic effect potentially through the inhibition of geranylgeranylation.

### The effects of atorvastatin on Sertoli cells partially depend on the Rac1/AP1/MMPs pathway

**Fig. 5:**
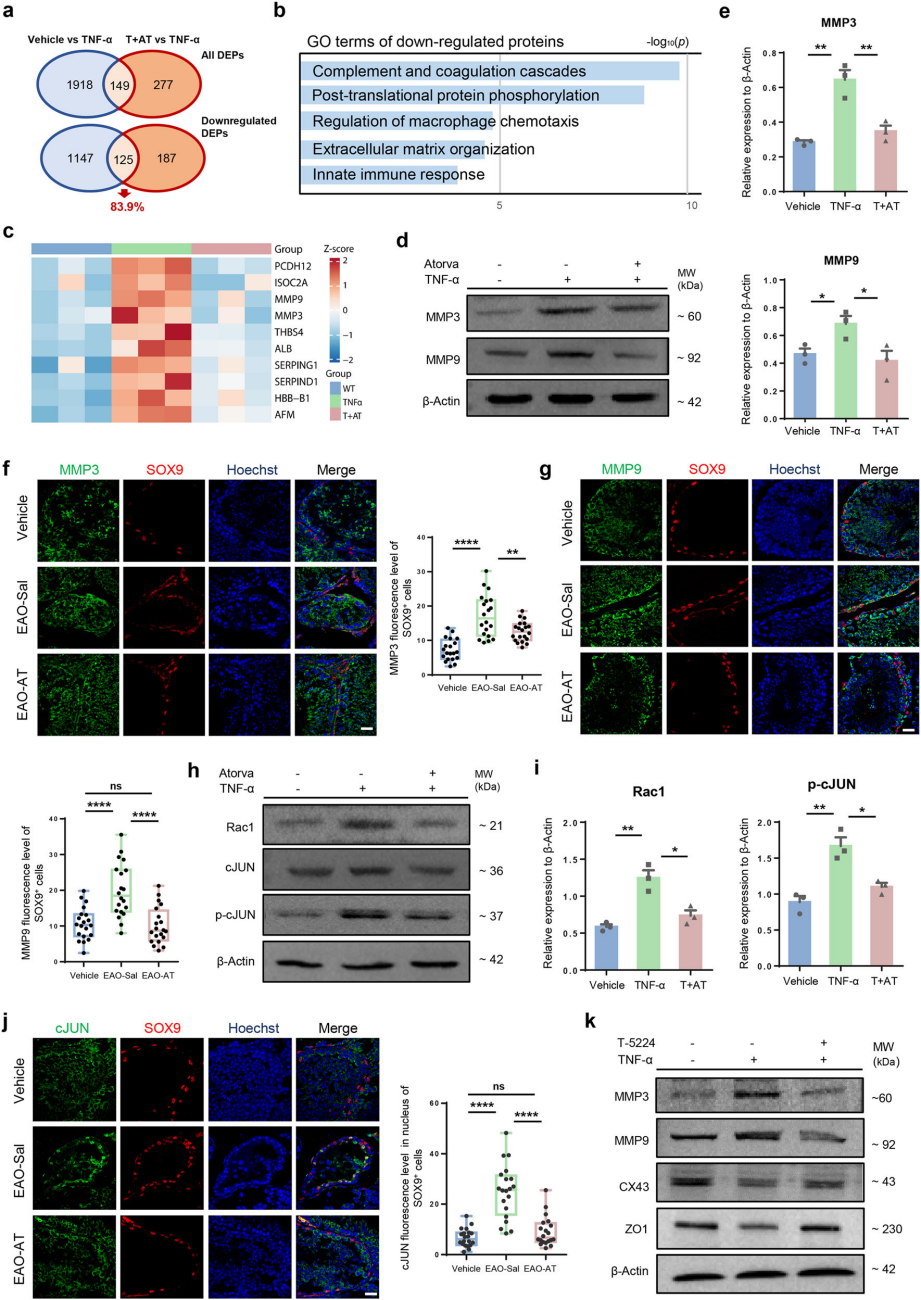
The effects of atorvastatin on Sertoli cells partly depend on Rac1/AP1/MMPs pathway. **a** Venn diagram of differential expressed proteins (DEPs) between groups. **b** Enriched GO terms of the co-downregulated proteins in Vehicle and T + AT groups compared with TNF-α group. **c** Heatmap showing the top 10 co-downregulated proteins according to the fold change between T + AT group and TNF-α group. **d** Western blot images of MMP3 and MMP9 in TM4 Sertoli cells from Vehicle, TNF-α and T + AT groups. **e** Grayscale value analysis of western blot for MMP3 and MMP9 in TM4 Sertoli cells from Vehicle, TNF-α and T + AT groups, *n* = 3. **f**, **g** Immunofluorescence of MMP3 and MMP9 (green) co-stained with SOX9 (red) in Vehicle, EAO-Sal and EAO-AT groups and quantitative analysis for immunofluorescence intensity of MMP3 and MMP9 in SOX9^+^ Sertoli cells from 20 randomly selected fields. SOX9 labeled Sertoli cells. Scale bar, 10 μm. *n* = 4 testis samples per group. **h** Western blot assay of Rac1, cJUN and Phospho-cJUN (p-cJUN) in TM4 Sertoli cells from Vehicle, TNF-α and T + AT groups. **i** Grayscale value analysis of western blot for Rac1 and p-cJUN in TM4 Sertoli cells from Vehicle, TNF-α and T + AT groups, *n* = 3. **j** Immunofluorescence of cJUN (green) co-stained with SOX9 (red) in Vehicle, EAO-Sal and EAO-AT groups and quantitative analysis for immunofluorescence intensity of cJUN in the nucleus of SOX9^+^ Sertoli cells from 20 randomly selected fields. SOX9 labeled Sertoli cells. Scale bar, 10 μm. **k** Western blot images of MMP3, MMP9, CX43 and ZO1 in TM4 Sertoli cells after T-5224 treatment. Data are presented as mean ± SEM, * *P* < 0.05, ** *P* < 0.01, **** *P* < 0.0001, ns indicates no statistical significance.

To further investigate the specific molecular pathways involved in the improvement of BTB-associated protein expression by atorvastatin, three groups of TM4 cells with different treatments were harvested for TMT-labeled quantitative proteomics. The principal component analysis of proteomic data demonstrated the reproducibility within each group (Fig. S5a). KEGG pathway enrichment analysis revealed that compared with the TNF-α group, the differentially expressed proteins (DEPs) of the Vehicle group and the T + AT group were both associated with processes such as focal adhesion, extracellular matrix–receptor interaction, and TNF signaling pathway, which were relevant to the BTB and spermatogenic microenvironment (Fig. S5b). We then overlapped the DEPs between the Vehicle group and the T + AT group with the TNF-α group. Among these, 125 proteins (83.9%) were significantly co-downregulated, suggesting that these proteins potentially play a crucial role in maintaining the function of TM4 Sertoli cells, as they exhibit a similar trend to the vehicle group (Fig. 5a). GO analysis of these co-downregulated proteins showed that they were related to pathways such as complement and coagulation cascades, regulation of macrophage chemotaxis and extracellular matrix organization, which was consistent with KEGG analysis to some extent (Fig. 5b).

Next, we focused on the specific co-downregulated differential proteins and ranked them according to the fold change between T + AT group and TNF-α group. The top 10 proteins were shown (Fig. 5c), among which we found matrix metallopeptidases 3 and 9 (MMP3 and MMP9) of the metalloproteinase family were significantly elevated in TNF-α-induced TM4 cells (3.35-fold elevation of MMP3, 5.21-fold elevation of MMP9). However, after atorvastatin intervention, their expression was markedly reduced (0.27-fold reduction of MMP3, 0.24-fold reduction of MMP9). Previous studies have reported that MMPs in the testis participate in the maintenance of homeostasis in the spermatogenic microenvironment, and excessive MMPs can hydrolyze intercellular junction proteins and inhibit their expression, leading to the BTB disruption [32, 33]. Therefore, we speculated that the effect of atorvastatin might be achieved by inhibiting the TNF-α-induced elevation of MMPs. Accordingly, we confirmed the altered protein levels of MMP3 and MMP9 in each group by western blot analysis (Fig. 5d, e). More importantly, we further verified that atorvastatin administration could significantly reduce the elevated levels of MMP3 and MMP9 proteins in Sertoli cells of EAO mice that were treated with atorvastatin (Fig. 5f, g).

Further GO analysis revealed that Rho GTPase activation of formins was enriched in pathways significantly upregulated in TM4 cells after TNF-α induction (Fig. S5c), whereas downregulation of Rho protein signal transduction could be enriched after atorvastatin intervention (Fig. S5d). In particular, Proteomic data showed Rac1 and RhoC, members of the Rho GTPase family, were significantly elevated under inflammation and decreased to a certain extent (Fig. S5e). Additionally, it has been reported that Rac1 could regulate the expression of MMPs by activating the transcription factor AP1/cJUN [34, 35]. Considering that both Rac1 and AP1 are the downstream molecules involved in statins’ inhibition of isoprenylation modifications [16] and based on our results with isoprenylation inhibitors in Fig. 4g, h, we thus hypothesized that atorvastatin might attenuate inflammation-induced damage to the BTB and disturbances in the spermatogenic niche via the Rac1 /AP1/MMPs pathway. To test this, we first demonstrated that atorvastatin significantly reduced the elevated level of Rac1 and the degree of AP1/cJUN activation (phosphorylated cJUN) in Sertoli cells induced by TNF-α (Fig. 5h, i), which tendency was in agreement with the alteration of MMP3/9 level. Consistently, the AP1 activation (increased cJUN nuclear localization) in Sertoli cells could be effectively inhibited by atorvastatin intervention in EAO mice (Fig. 5j). In addition, we found that Rac1 inhibitor 1A-116 could markedly inhibit AP1/cJUN activation by decreasing p-c-JUN level, as well as downregulated MMP3 and MMP9 expressions upon TNF-α induction (Fig. S6a, b), suggesting that its similar role as atorvastatin in suppressing the AP1 activation and reducing MMPs level. On the contrary, under inflammatory conditions, Rac1 overexpression counteracted the therapeutic effects of atorvastatin in downregulating p-c-JUN and MMPs expression, thereby impeding restoration of BTB-associated proteins (Fig. S6c–f). Furthermore, treatment with AP1/cJUN complex inhibitor T-5224 exhibited a decrease in MMPs and an increase in BTB-associated proteins (Figs. 5k and S6g), reflecting the similar effects with atorvastatin. These results suggested that atorvastatin might enhance the compromised expression of BTB-associated proteins via inhibiting Rac1/AP1/MMPs pathway in Sertoli cells, thereby benefiting BTB integrity.

### Atorvastatin restores spermatogenesis in LPS-induced chronic orchitis mice model

**Fig. 6:**
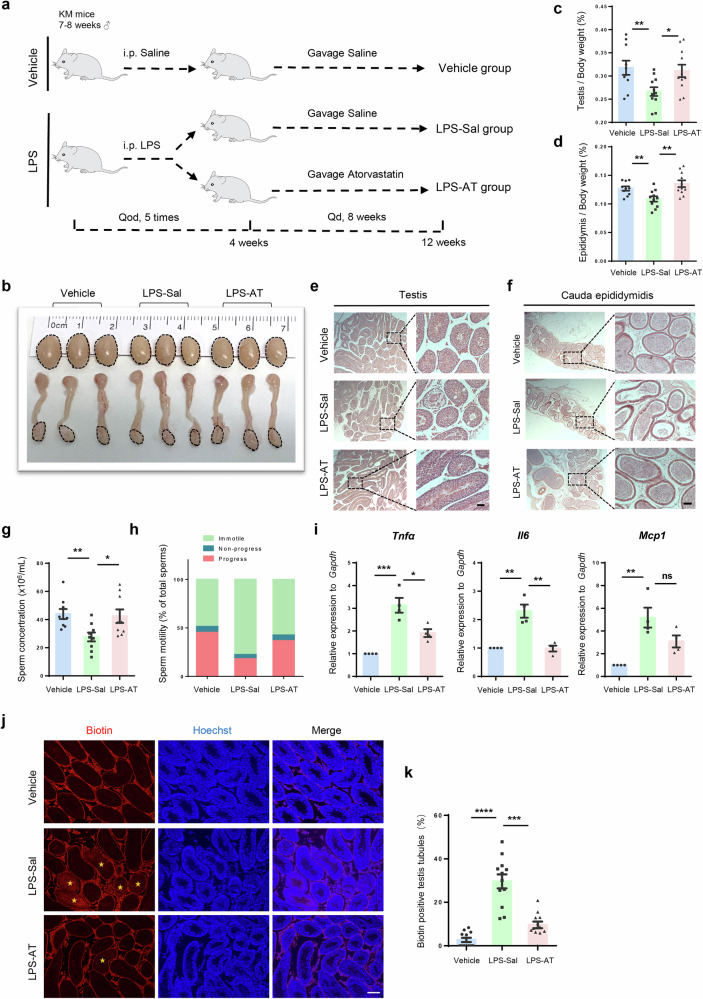
Atorvastatin improves phenotypes of LPS-induced chronic orchitis mouse models. **a** Schematic illustration of the experimental design for the gavage administration of Atorvastatin in LPS-induced mice. **b** Bright field images of mouse testis and epididymis in Vehicle, LPS-Sal and LPS-AT groups. **c**, **d** Coefficient of testis (testis/body weight) and epididymis (epididymis/body weight) in Vehicle, LPS-Sal and LPS-AT groups, *n* = 10. Histological sections of testis (**e**) and cauda epididymidis (**f**) in Vehicle, LPS-Sal and LPS-AT groups, stained by H&E staining, dotted box represents the enlarged area shown. Scale bar, 400 μm (left), 200 μm (right). **g**, **h** Sperm concentration and sperm motility in Vehicle, LPS-Sal and LPS-AT groups, *n* = 9. **i** Relative mRNA levels of *Tnfα*, *Il6*, and *Mcp1* in the testis from Vehicle, LPS-Sal and LPS-AT groups measured by quantitative PCR. *Gapdh* (Glyceraldehyde-3-phosphate dehydrogenase) serves as internal reference, *n* = 4. **j**, **k** Immuno-fluorescence of biotin (red) and biotin-positive seminiferous tubules percentage in testis of Vehicle, LPS-Sal and LPS-AT groups from 12 randomly selected fields. Yellow asterisks indicate biotin-positive seminiferous tubules. Scale bar, 50 μm. *n* = 4 testis samples per group. Data are presented as mean ± SEM, *P < 0.05, **P < 0.01, ***P < 0.001, ****P < 0.0001, ns indicates no statistical significance.

In addition to the mouse models of chronic orchitis induced by autoimmune processes, we also generated a model of infection induced by the continual administration of a low dose of lipopolysaccharide (LPS), which mimics the chronic orchitis progressed from acute inflammation [36, 37] (Fig. 6a). Similar to the EAO model, 8-week intervention with atorvastatin could effectively alleviate the symptoms of orchitis in LPS-induced mice, including mitigating the testis and epididymis atrophy and markedly increasing the coefficients of testis and epididymis (Fig. 6b–d). HE staining showed that atorvastatin can significantly ameliorate the reproductive pathological damage, and the vacuolation of seminiferous epithelia, germ cell exfoliation caused by LPS (Fig. 6e). Consistently, the significantly reduced sperm density in the cauda epididymidis was visibly ameliorated after atorvastatin treatment (Fig. 6f). Additionally, we collected the cauda epididymidis of mice from three groups for sperm parameter assays. Consistent with the effects in EAO mice, atorvastatin notably attenuated the reduction of sperm concentration and sperm motility caused by LPS and enhanced sperm quality (Fig. 6g, h).

We next found that the gene expression of inflammatory factors, including TNF-α, IL-6, and MCP-1, was downregulated (Fig. 6i) along with reduced macrophage numbers (Fig. S7a, b), which indicates the inflammation level were ameliorated by atorvastatin. Furthermore, biotin tracing assay demonstrated that atorvastatin restored the integrity of BTB in LPS-induced model mice (Fig. 6j, k). Together, these results suggest that atorvastatin showed a potent therapeutic effect in response to both infectious and noninfectious causes of chronic orchitis.

### Atorvastatin re-constructs BTB and increases germ cell production in human testis in vitro

**Fig. 7:**
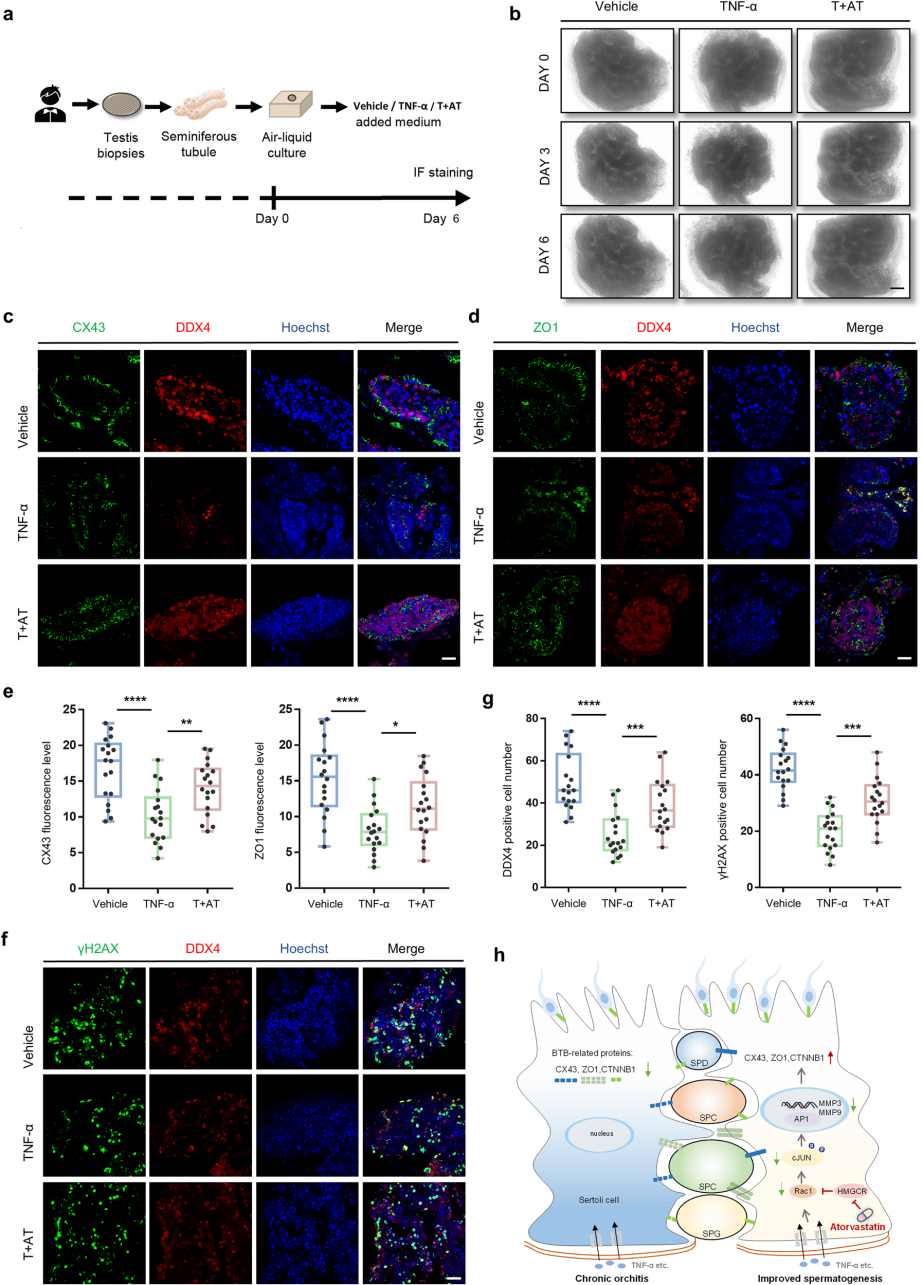
Atorvastatin re-establishes BTB protein expression and increases germ cells in human cultured testis. **a** Schematic illustration of human seminiferous tubule culture conditions in vitro. **b** Bright field diagram of human cultured testis in Vehicle, TNF-α (medium supplemented with TNF-α) and T + AT (medium supplemented with TNF-α and atorvastatin) groups. Scale bar, 10 mm. **c**, **d** Immunofluorescence of CX43 and ZO1 (green) co-stained with DDX4 (red) in human testis from 3 groups. Scale bar, 10 μm. **e** Quantitative analysis for immunofluorescence intensity of CX43 and ZO1 from 18 randomly selected fields, *n* = 4 human testis samples per group. **f**, **g** Immunofluorescence of γH2AX (green) co-stained with DDX4 (red) in human testis from Vehicle, TNF-α, and T + AT groups and statistical histogram of the number of germ cells and spermatocytes from 18 randomly selected fields. DDX4 labeled germ cells, DDX4 and γH2AX co-staining labeled spermatocytes. Scale bar, 10 μm. *n* = 4 human testis samples per group. **h** Hypothetical schematic diagram depicting the role of atorvastatin in protecting the BTB from TNF-α-induced impairment through suppressing the Rac1-AP1-MMPs signaling axis by inhibiting HMGCR in Sertoli cells. SPG spermatogonia, SPC spermatocyte, SPD spermatid. Data are presented as mean ± SEM, *P < 0.05, **P < 0.01, ***P < 0.001, ****P < 0.0001.

To expand our findings to clinical relevance, we employed a human testis culture method that could maintain tissue cell identity for 6 days [38]. Then, the effect of atorvastatin was further examined in TNF-α-induced inflammation of cultured human testis tissues, which morphology remained unchanged during the culture period (Fig. 7a, b). Consistent with our previous findings in mice models, atorvastatin could effectively restore the expression of BTB-associated proteins, including CX43 and ZO1, in human testis tissues after TNF-α induction (Fig. 7c–e). More importantly, the concomitant addition of atorvastatin effectively reversed the loss of DDX4-positive germ cells and γH2AX-positive spermatocytes in cultured human testis tissues caused by TNF-α (Fig. 7f, g). These results suggested that atorvastatin played a similar role in improving the BTB and protecting germ cells in inflammation-induced human testicular tissues, indicating its potential for treating chronic orchitis. The hypothetical schematic diagram of our study is shown in Fig. 7h.

## Discussion

Orchitis is a significant factor contributing to male infertility, accounting for 6–15% of male infertility cases. Even after pathogens are cleared, inflammatory responses and disruptions to the testicular niche persist, leading to chronic orchitis and impaired spermatogenesis. However, there are no standardized clinical treatment guidelines for chronic orchitis. In the present study, we have found that atorvastatin could alleviate the testicular inflammation and enhance spermatogenesis in both infectious and noninfectious chronic orchitis models. Our mechanistic studies suggested that atorvastatin might act through HMGCR-Rac1/AP1/MMPs axis to restore the integrity of the BTB, which might benefit fertility. More importantly, atorvastatin also re-established the BTB and increased germ cell numbers in cultured human testis tissue. Thus, our findings provided a novel strategy for the treatment of chronic orchitis with great clinical translational potential.

In clinical practice, experience-based empirical medications for chronic orchitis include glucocorticoids and some NSAIDs [5] often yields suboptimal results. Specifically, glucocorticoid therapy does not improve the chances of natural conception, resulting in the demands of in vitro fertilization (IVF) or intracytoplasmic sperm injection for these patients, and these drugs often bring systemic side effects [39–41]. Additionally, NSAIDs treatment could only temporarily increase sperm counts and reduce the number of leukocytes in semen. Once the treatment is stopped, azoospermia and leukocytospermia will recur [42]. Recent therapeutic agents for orchitis, included melatonin and PTH1R agonist have demonstrated effectiveness in reducing the inflammatory levels and cell apoptosis, yet whether they could improve spermatogenesis or fertilizing capability remain elusive because lack of comprehensive analysis of spermatogenic cell types and fertility [43, 44]. Of note, our study indicated that atorvastatin could improve testicular and epididymal coefficients, reduce pathological damage to the reproductive system, enhance sperm quantity and quality, and indeed promote spermatogenesis in two different types of chronic orchitis model mice. Additionally, it significantly improved the fertility of EAO mice, while simultaneously assessing its safety at the effective dose. This offers substantial experimental evidence for the clinical application of statins, particularly atorvastatin, in the treatment of chronic orchitis. More importantly, the effects of atorvastatin in enhancing sperm quality persisted for up to 3 months post-drug withdrawal, thereby rendering it more suitable for the treatment of patients with infertility.

In our previous study, statins significantly promote the self-renewal of murine and primate SSCs [20]. The current study further indicated that atorvastatin treatment could increase germ cells at all stages, including spermatogonia, spermatocytes, and spermatids. These findings suggested that the promoting effect of atorvastatin on spermatogenesis in chronic orchitis model mice was not limited to a specific germ cell type, but rather a more general effect, which is in line with our subsequent observations regarding the overall improvement of the spermatogenic microenvironment by atorvastatin. Here, we provided robust evidence that atorvastatin enhanced BTB by suppressing HMGCR-Rac1/AP1/MMPs axis using various methods, including shRNA knockdown or specific inhibitors of the downstream pathway. As Sertoli cells are known as playing a central role in maintaining spermatogenesis [45], we thus speculated that Sertoli cells play a key role in the administration of atorvastatin, considering their vital function in the testis. Interestingly, atorvastatin only moderately, rather than significantly, improved the decreased testosterone levels in EAO mice. We speculated that this might be due to the fact that statins could inhibit the synthesis of cholesterol, which was a prerequisite substance for testosterone synthesis [46], and therefore had a limited effect on Leydig cells under inflammation.

MMPs are homeostasis regulators of the BTB, and the disturbance of BTB is often accompanied by significant changes in MMPs gene expression [32]. Studies have reported that inflammatory factors such as TNF-α could promote the expression of MMPs in different tissues [47, 48]. In our study, we found that atorvastatin significantly repressed the upregulation of MMP3 and MMP9 in Sertoli cells under inflammation. In addition, the Rac1 and AP1/cJUN, which were reported to participate in MMP-mediated matrix remodeling and angiogenesis [35, 49], were found to be repressed after atorvastatin treatment in our study. Moreover, the knockdown of HMGCR and specific inhibitors of Rac1 and AP1/cJUN could mimic the effect of atorvastatin. All these results indicated that atorvastatin could protect the BTB from inflammation-induced impairment through suppressing the Rac1-AP1-MMPs axis. However, we could only partially restore the BTB and recover fertility of chronic orchitis mouse models, suggesting that inflammation might induce BTB damage and impair spermatogenesis via other factors. Furthermore, despite we found atorvastatin improved BTB by regulating Rac1, we could not determine whether other small GTPase family proteins were also involved in the destruction of BTB in the context of chronic orchitis. Further investigation is needed to explore the underlaying mechanism and more specific and useful treatment for chronic orchitis patients is to be exploited.

In summary, our study uncovers a significant therapeutic effect of atorvastatin in murine models of both infectious and non-infectious chronic orchitis through restoring BTB integrity by suppressing the mevalonate pathway and the Rac1/AP1/MMPs axis of Sertoli cells, thereby benefiting spermatogenesis. Notably, atorvastatin re-established the expression of BTB-associated proteins and increased germ cell numbers in cultured human testis tissues under inflammation-induced conditions. Therefore, we propose that atorvastatin represents a promising approach for improving fertility in chronic orchitis patients, providing an important basis of its clinical application in treating chronic orchitis-associated infertility.

## Materials and methods

### Animal models

C57BL/6J and KM mice (7–8 weeks old) were procured from Southern Medical University Laboratory Animal Center and were divided randomly into three groups. All the mice were housed on a 12 h light/dark cycle and given food and water ad libitum. All animal care and experimental procedures were approved by the Institutional Animal Care and Use Committee of Southern Medical University, China (ethical code: L2016149) and carried out in accordance with the National Institutes of Health guide for the care and use of Laboratory animals. For the induction of EAO [21, 50] male C57BL/6J mice were actively immunized with testicular homogenate (TH) in complete Freund’s adjuvant (CFA; F5881, Sigma-Aldrich, Saint Louis, MO, USA) as follows: testes from mice were collected, decapsulated, digested and homogenized at a ratio of 1:1 in saline solution to obtain TH and then mixed 1:1 with CFA to get a total volume of 200 µl for each mouse and subcutaneous injected (s.c.) three times every 14 days. Every immunization was accompanied by intraperitoneal injection (i.p.) of 100 ng Bordetella pertussis toxin (BPT; P7208, Sigma-Aldrich) dissolved in 100 μL PBS (pH 7.6). After 4 weeks from the first injection, animals were divided randomly into two groups and administered by gavage with atorvastatin (T3116, TargetMol, USA) solution (EAO-AT group) or saline (EAO-Sal group) for 8 weeks. For LPS models [51], male KM mice received i.p. injection of lipopolysaccharide (LPS; L2630-10MG, Sigma Aldrich) at 3 mg/kg every 2 days for five times. After 4 weeks from the first injection, animals were administered by gavage with 10 mg/kg atorvastatin solution (LPS-AT group) or saline (LPS-Sal group) for 8 weeks. Age-matched mice administered by saline injection and gavage were recognized as Vehicle group. An online sample size calculator (https://clincalc.com/stats/samplesize.aspx) was used for sample size estimate. The mice of each group were randomly assigned according to the random number table before treatment, and no blinding was done.

### Histopathological analysis

All testicular tissues from mice and human were immersed in 4% paraformaldehyde (P0099, Beyotime Biotechnology, Shanghai, China) for 24 h at 4 °C. Subsequently, the tissues were dehydrated through an ethanol gradient (70%, 80%, 90%, 100%I, 100%II, xylene I, and xylene II) to remove water and embedded in paraffin. Sections of 5–7 μm thickness were cut for analysis. Then, dewaxed and rehydrated paraffin sections were stained with hematoxylin and eosin (H&E) and dehydrated using ethanol and xylene and finally mounted using resin for routine pathological examination. The tissue images were captured under an OLYMPUS BX51 microscope.

### Immunofluorescence staining

Tissue sections were dewaxed, rehydrated, and subjected to antigen retrieval. Briefly, samples were blocked with 5B (PBS containing 5% BSA) for 60 min at room temperature and incubated overnight with primary antibody. The following day, after PBS washing for three times, samples were incubated with secondary antibody at room temperature for 1 h. Finally, sections were rinsed in PBS for three times and sealed with an anti-fluorescence attenuation quenching agent (S2100, Beijing Solarbio Science & Technology Co., Ltd, China). Fluorescent images were captured under a ZEISS LSM880 confocal microscope and the mean fluorescence intensity was analyzed using the Image J software. The primary antibodies used in this study were listed: mouse anti-DDX4 (ab27591, Abcam, 1:600, Cambridge, UK), rabbit anti- DDX4 (ab13840, Abcam, 1:600), mouse anti-PLZF (sc-28319, Santa Cruz Biotechnology, 1:200, Santa Cruz, CA, USA), rabbit anti-SYCP3 (ab15093, Abcam, 1:500), mouse anti-γH2AX (ab26350, Abcam, 1:500), mouse anti-CREM (Santa Cruz Biotechnology, sc-390426, 1:50), rabbit anti-F4/80 (70076S, Cell Signaling Technology, Danvers, MA, USA), rabbit anti-SOX9 (Millipore, AB5535, 1:400, Billerica, MA, USA), mouse anti-SOX9 (67439-1-Ig, Proteintech, 1:200, Chicago, IL, USA), rabbit anti-CX43 (ab217676, Abcam, 1:200), rabbit anti-ZO1 (ab221547, Abcam, 1:400).

### Sperm parameters detection

Male mice were euthanized by cervical dislocation and cauda epididymis was dissected and minced in M2 medium (M7167, Sigma Aldrich) to release and disperse sperm. Sperm concentration and motility were automatically assessed using the IVOS II (Hamilton Thorne Inc., Beverly, MA, USA) computer-assisted sperm analysis system.

### In vitro fertilization (IVF)

IVF was performed according to previously described methods [38]. Briefly, Spermatozoa were collected by cutting the cauda epididymis and squeezing it gently in 200 μL HTF solution (M1130, Easy Check, Nanjing, China) covered with mineral oil, allowing the sperm to swim out freely and left for 1 h in a 34 °C, 5% CO_2_ incubator for capacitation. Cumulus-Oocyte Complexes (COCs) were disassociated from oviductal ampullae and placed into another 200 μL HTF droplet. Then capacitated sperm were added to the HTF drop containing COCs at a final concentration of 1 × 10^6^ sperm/mL. After 6 h incubation, HTF was replaced with KSOM medium (M1430, Easy Check) for further culture. The number of embryos at different developmental stages was counted under the microscope for statistical analysis.

### ELISA assay

The whole blood of mice was obtained by extracting the eyeball blood under anesthesia with pentobarbital sodium. The blood samples were stored at 4 °C overnight and then centrifuged at 1000 × *g* for 15 min at 4 °C. The clear serum of the upper layer was collected. Serum concentration of hormones or cytkines including Testosterone (SEKSM-0003, Beijing Solarbio Science & Technology Co., Ltd); TNF-α (SEA133Mu, Cloud-Clone, Wuhan, China); IL-6 (SEA079Mu, Cloud-Clone) and MCP-1 (SEA087Mu, Cloud-Clone) were evaluated using the ELISA kit according to the manufacturer’s instructions.

### RNA extraction and quantitative real-time PCR (RT-qPCR)

Total RNA was extracted from cultured cells using Trizol reagent (DP424, TIANGEN, Beijing, China). Reverse transcription was carried out using Vazyme RT reagent kit (R123-01, Vazyme, Nanjing, China). Real-time qPCR was carried out using SYBR Green Master Mix (Q511-02, Vazyme) in a qPCR instrument (Veriti, ABI). The expression levels of target genes were normalized to the expression of the housekeeping gene *Gapdh* and were calculated using the 2^−△△Ct^ method. The primer sequences used in this study are listed in Supplementary Table 1.

### Acquisition and processing of Single-cell RNA sequencing data

The dataset GSM5563669 was downloaded from the GEO database (www.ncbi.nlm.nih.gov/geo), which was used in the original article as EAO mice [23]. Cells were integrated and filtered under Seurat 4.0.5, (nFeature_RNA > 200, nFeature_RNA < 7000, percent.mt <20, and min cells = 3), resulting in a filtered expression matrix containing 53,952 genes and 6929 cells. The gene-expression measurements for each cell were normalized with the parameter “scale factor = 100,000”. Subsequently, the top 2000 highly variable genes and 1–50 principle components (PCs) were used in the cluster analysis. The uniform manifold approximation and projection (UMAP) technique was used to visualize the dataset by choosing 1–10 PCs in the cluster analysis. The cell types were then identified by plotting marker genes with the function Feature Plot. Based on the unbiased clustering results and the expression of known characteristic genes, six cell subpopulations were defined, including spermatogonia (Sg), spermatocytes (Sc), spermatids (Sd), Sertoli cells (St), Leydig cells (Lc), and immune cells (Im). For differentially expressed genes, the default parameters of the FindMarkers function from Seurat were used, which is the Wilcoxon rank-sum test with log2fold change (FC) threshold of 0.25 and min.pct of 0.1 with *P* value < 0.05.

### Cell–cell communication analysis

The expression matrices of scRNA-seq data were processed using the Seurat package. Cell–cell communication networks were identified and visualized using the CellChat (V1.5.0) package, following the standard workflow [52].

### Transmission electron microscopy

Testicular tissues of mice from different groups were fixed in 2.5% glutaraldehyde and divided into 0.5 × 0.5 × 1 cm pieces, rinsed in phosphate buffer solution (PBS) and fixed in 1% osmium acid for 1 h. Dehydrated through a graded ethanol series, the samples were embedded in a resin and sliced [53, 54]. Ultrathin sections were stained with 1% uranyl acetate and lead citrate and imaged using a transmission electron microscope (Hitachi H-7500).

### Integrity detection of blood-testis barrier

The integrity of BTB was detected using a biotin tracer as previously described [38]. To be specific, the male mice from each group were anesthetized with pentobarbital sodium at a dose of 0.08 mg/kg, then 20 μl EZ-Link Sulfo-NHS-LC-Biotin (21335, ThermoFisher, Waltham, MA, USA; 10 mg/mL in PBS containing 1 mM CaCl_2_) was injected into the testicular stroma. 30 min later, the mice were euthanized by cervical dislocation, and the testicles were collected. Subsequently, biotin penetration was observed by immunofluorescence staining using Streptavidin CY^TM^3 conjugate (SA1010, Invitrogen, Waltham, MA, USA) to detect the integrity of BTB in mice.

### Cell culture and reagent

The TM4 (immortalized mouse Sertoli cells) and HEK293T cell lines were purchased from Procell Life Science & Technology Co., Ltd. (Wuhan, China). All the cells were cultured in DMEM basic medium (12100061, Gibco, Grand Island, NY, USA) supplemented with 10% fetal bovine serum (SE100-011, VISTECH, Australia) and 1% penicillin and streptomycin (15140122, Gibco) in 37 °C humidified incubators with 5% CO_2_. Other reagents used in this study were listed as follows: Dimethyl sulfoxide (DMSO; D2650, Sigma Aldrich), atorvastatin (T3116, TargetMol), TNF-α recombinant protein (HZ-1014, Proteintech), FTI-277 (T2700-1, TargetMol), GGTI-298 (T6844-2, TargetMol), 1A-116 (T14004, TargetMol), T-5224 (T5416, TargetMol).

### Plasmid transfection and construction of stable cell lines

Plasmids encoding shRNAs or 3xFlag-Rac1 were obtained from Tsingke Biotechnology. HEK293T cells at a density of about 80% were transfected with *Hmgcr*-shRNA1-4, shRNA-NC and packaging plasmids, psPAX2 and pMD2.G or 3xFlag-Rac1, ovexpression-NC, and packaging plasmids. Medium containing Lentiviral particle was collected 48 h after transfection. Harvested media mixed 1:1 with fresh medium were added to TM4 Sertoli cells, and stable cell lines of *Hmgcr*-knockdown or 3xFlag-Rac1 overexpression were achieved by puromycin (ST1450, Beyotime Biotechnology, Shanghai, China) selection. The following shRNAs were used in this study: mouse *Hmgcr* shRNA-1: 5′-CCGGGCTTGGGTCCAAGTACATTCTCTCGAGAGAATGTACTTGGACCCAAGCTTTTTT-3′, mouse *Hmgcr* shRNA-4: 5′-CCGGGCAGGACGCAACCTCTATATCCTCGAGGATATAGAGGTTGCGTCCTGCTTTTTT-3′, mouse shRNA-NC: 5′- GGTTCTCCGAACGTGTCACGT-3′.

### Western blotting

TM4 cells were lysed in the RIPA buffer (R0010, Beijing Solarbio Science & Technology Co., Ltd). Protein samples were separated by SDS-PAGE using polyacrylamide gels and then transferred to polyvinylidene difluoride PVDF membranes (05039A, PALL, Port Washington, NK, USA). After blocking with 5% Milk-TBST, PVDF membranes were incubated with specific primary antibodies in 5% Milk-TBST at 4 °C overnight then washed and incubated in corresponding horseradish peroxidase conjugated secondary antibodies for 60 min at room temperature. Immunoreactive bands were visualized and detected by enhanced chemiluminescence (ECL; G3308, GBCBIO Technologies, Guangzhou, China). The primary antibodies used in this study were listed: rabbit anti-HMGCR (A19063, ABclonal, 1:1000), rabbit anti-CX43 (26980-1-AP, Proteintech, 1:1000), rabbit anti-ZO1 (21773-1-AP, Proteintech, 1:1000), rabbit anti-CTNNB1 (ab32572, Abcam, 1:1000), rabbit anti-MMP3 (17873-1-AP, Proteintech, 1:1200), rabbit anti-MMP9 (10375-2-AP, Proteintech, 1:1200), rabbit anti-cJUN (ab32137, Abcam, 1:4000), rabbit anti-p cJUN (9261S, Cell Signaling Technology, 1:1000), rabbit anti-Rac1 (24072-1-AP, Proteintech, 1:1000). The 180 kDa Plus Prestained Protein Marker (MP201, Vazyme) was used as protein marker in all WB experiments.

### Proteomic detection

The data-independent acquisition (DIA) quantitative proteomics detection and analysis were performed by the Shanghai BIOPROFILE Technology Co., Ltd (Shanghai, China). In detail, the protein of TM4 Sertoli cell samples was first extracted using SDT lysis buffer (4% SDS, 100 mM DTT, 100 mM Tris-HCl pH 8.0). Samples were boiled for 3 min and further ultrasonicated. Undissolved cellular debris was removed by centrifugation at 16,000 × *g* for 15 min. The supernatant was collected and quantified with a BCA Protein Assay Kit (BeyoTime, China). Protein digestion was performed with FASP method described by Wisniewski, Zougman et al. [55]. Briefly, the detergent, DTT, and IAA in UA buffer were added to block reduced cysteine. Finally, the protein suspension was digested with trypsin (Promega) at ratio 50:1 overnight at 37 °C. The peptide mixtures were collected by centrifugation at 16,000 × *g* for 15 min and desalted with C18 StageTip for further LC-MS analysis. The concentrations of re-dissolved peptides were determined with OD280 by Nanodrop One device (Thermo, USA). LC–MS/MS were performed on an Orbitrap Astral mass spectrometer coupled with Vanquish Neo UHPLC system (Thermo Fisher Scientific). Peptides from each sample were loaded into a column (50 cm Low-Load µPAC™ Neo HPLC Column, Thermo Scientific) at a flow rate of 2.2 μL/min. The RP−HPLC mobile phase A was 0.1% formic acid in water, and B was 0.1% formic acid in 80% acetonitrile. Peptide were eluted over 8 min with a linear gradient of buffer B at 1.25 μL/min. The linear gradient was set as follows: 0–0.1 min, linear gradient from 4 to 6% buffer B; 0.1–1.1 min, linear gradient from 6% to 12% buffer B; 1.1–12 min, linear gradient from 12 to 25% buffer B; 12–18 min, linear gradient from 25 to 45% buffer B; 18.0–18.5 min, linear gradient from 45 to 99% buffer B; 18.5–24 min, buffer B maintained at 99%. The eluted peptides were analyzed on an Orbitrap Astral mass spectrometer. The DIA method consisted of a survey scan from 380 to 980 m/z at resolution 240,000 with AGC target of 500% and 5 ms injection time. The DIA MS/MS scans were acquired by Astral from 150 to 2000 m/z with 2 m/z isolation window and with AGC target of 500% and 3 ms injection time. Normalized collision energy was set 25 and cycle time was 0.6 s. The spectra of full MS scan and DIA scan were recorded in profile and centroid type, respectively.

### Proteomic analysis

The DIA MS data were analyzed using DIA-NN 1.8.1 [56]. MS data were searched against the Reference-Musmusculus (Mouse) [10090]-20230924. fasta, https://www.uniprot.org/taxonomy/10090. The trypsin was selected as the digestion enzyme. The maximal 1 missed cleavage sites and the mass tolerance of 10 ppm for precursor ions and 10 ppm for fragment ions were defined for database search. Carbamidomethylation of cysteines was defined as fixed modification, while acetylation of protein N-terminal, oxidation of Methionine was set as variable modifications for database searching. Maximum number of variable modifications was 1. Peptide length range was set from 7 to 30. Charge of peptide was from 1 to 4. The fragment ion m/z range was from 150 to 2000. The database search results were filtered and exported with <1% false discovery rate at peptide-spectrum-matched level, and protein level, respectively. For statistical analysis, proteins with fold change ≥1.2 as well as *P* < 0.05 were considered as DEPs. The list of DEPs is provided in Supplemental data.

### Human testicular tissue in vitro culture

The human testicular tissue samples were approved by the medical ethics committee of Southern Medical University (ethical code: NFEC-2019-219) and conducted in accordance with the Declaration of Helsinki on Ethical Principles for Medical Research. All samples were obtained with informed consent from the patients. The tissue was firstly washed twice using PBS and cut into about 2 mm small pieces, and then transferred to 1% agarose gel in the culture plate. The human testicular tissue culture basic medium was α-Minimum Essential Medium (αMEM; 32571036, Gibco) supplemented with 10% Knockout serum replacement (KSR; A3181502, Gibco), 0.1 mM NEAA (11140076, Gibco), 1 mM sodium pyruvate (11360070, Gibco), 2 mM Glutamax (35050061, Gibco), 1 mM penicillin streptomycin (15140122, Gibco) and 50 μM β-mercaptoethanol (21985023, Gibco). For the experimental groups, 50 ng/mL TNF-α or TNF-α combined 1 μM atorvastatin was added and then the plate was cultured at 35 °C humidified incubators with 5% CO_2_.

### Statistical analysis

All experiments were conducted in at least three biological replicates to ensure reproducibility. Data were analyzed and presented as mean ± SEM using GraphPad Prism 9. The Kolmogorov-Smirnov test and Levene’s test were used to test the normal distribution and homogeneity of variances successively. Statistical significance was shown as* *p* < 0.05, ** *p* < 0.01, *** *p* < 0.001, **** *p* < 0.0001, and ns means not significant. Unpaired two-tailed Student's *t* test and one-way ANOVA were used for comparisons between two groups or multiple groups, respectively.

## Supplementary information

Supplemental material-table and figures

Full and uncropped original Western blots

List of differentially expressed protein

## Data Availability

All data that support the findings of this study are available within the article and its supplementary materials, and raw data are available from the corresponding authors upon reasonable request.
