# Supplementary material for: Atorvastatin improves spermatogenesis in murine and in vitro human chronic orchitis models through restoring blood-testis barriers
Source: Cell Death Discov. 2025 Nov 6;11:505. doi: 10.1038/s41420-025-02749-6 (PMC12592372; doi:10.1038/s41420-025-02749-6)
Supplement: Supplementary file 1 — Supplemental material-table and figures [file 41420_2025_2749_MOESM1_ESM.docx]

**Supplementary Table 1. sequences of primer for quantitative real time PCR**

| Detected gene | Direction | primer squence |
| --- | --- | --- |
| *Gapdh* | Forward | TGTGTCCGTCGTGGATCTGA |
|  | Reverse | TTGCTGTTGAAGTCGCAGGA |
| *Tnfa* | Forward | TCGAGTGACAAGCCTGTAGC |
|  | Reverse | AAGGTACAACCCATCGGCTG |
| *Il6* | Forward | GAGGATACCACTCCCAACAGACC |
|  | Reverse | AAGTGCATCATCGTTGTTCATACA |
| *Mcp1* | Forward | TCTCACTGAAGCCAGCTCTC |
|  | Reverse | CGTTAACTGCATCTGGCTGAG |
| *Hmgcr* | Forward | AGCTTGCCCGAATTGTATGTG |
|  | Reverse | TCTGTTGTGAACCATGTGACTTC |

**
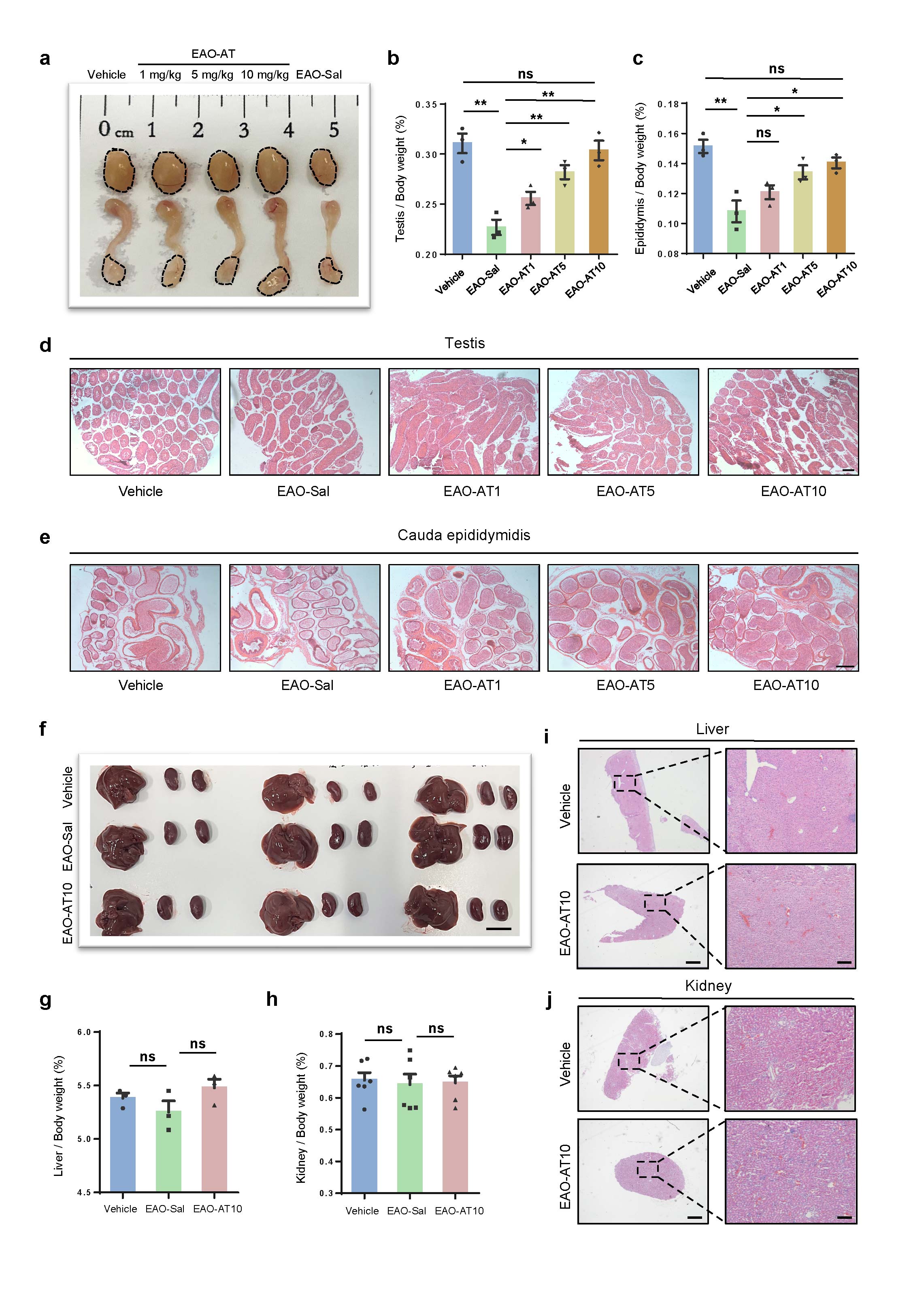
**

**Supplementary Fig. 1: Dose gradient screening and safety test of atorvastatin in EAO mice.** (a) Bright field images of mouse testis and epididymis in Vehicle, EAO mice treated with atorvastatin at 1/5/10 mg/kg (EAO-AT 1/5/10) and saline (EAO-Sal) groups. (b-c) Coefficient of testis (testis/body weight) and epididymis (epididymis/body weight) in Vehicle, EAO-Sal and EAO-AT 1/5/10 groups, *n* = 3. (d-e) H&E staining of sections of the testis and cauda epididymidis in Vehicle, EAO-Sal and EAO-AT 1/5/10 groups. Scale bar, 200 μm. (f) Bright field images of liver and kidney from Vehicle, EAO-Sal and EAO-AT10 groups. (g-h) Coefficient of liver (liver/body weight) and kidney (kidney/body weight) in Vehicle, EAO-Sal and EAO-AT10 groups, liver, *n* = 3; kidney, *n* = 6, samples from 3 mice. (i-j) H&E staining of liver and kidney sections in Vehicle and EAO-AT10 groups, dotted box represents the enlarged area shown. Scale bar, 500 μm (left), 200 μm (right). Data are presented as mean ± SEM, * *P <* 0.05, ** *P <* 0.01, ns indicates no statistical significance.

**
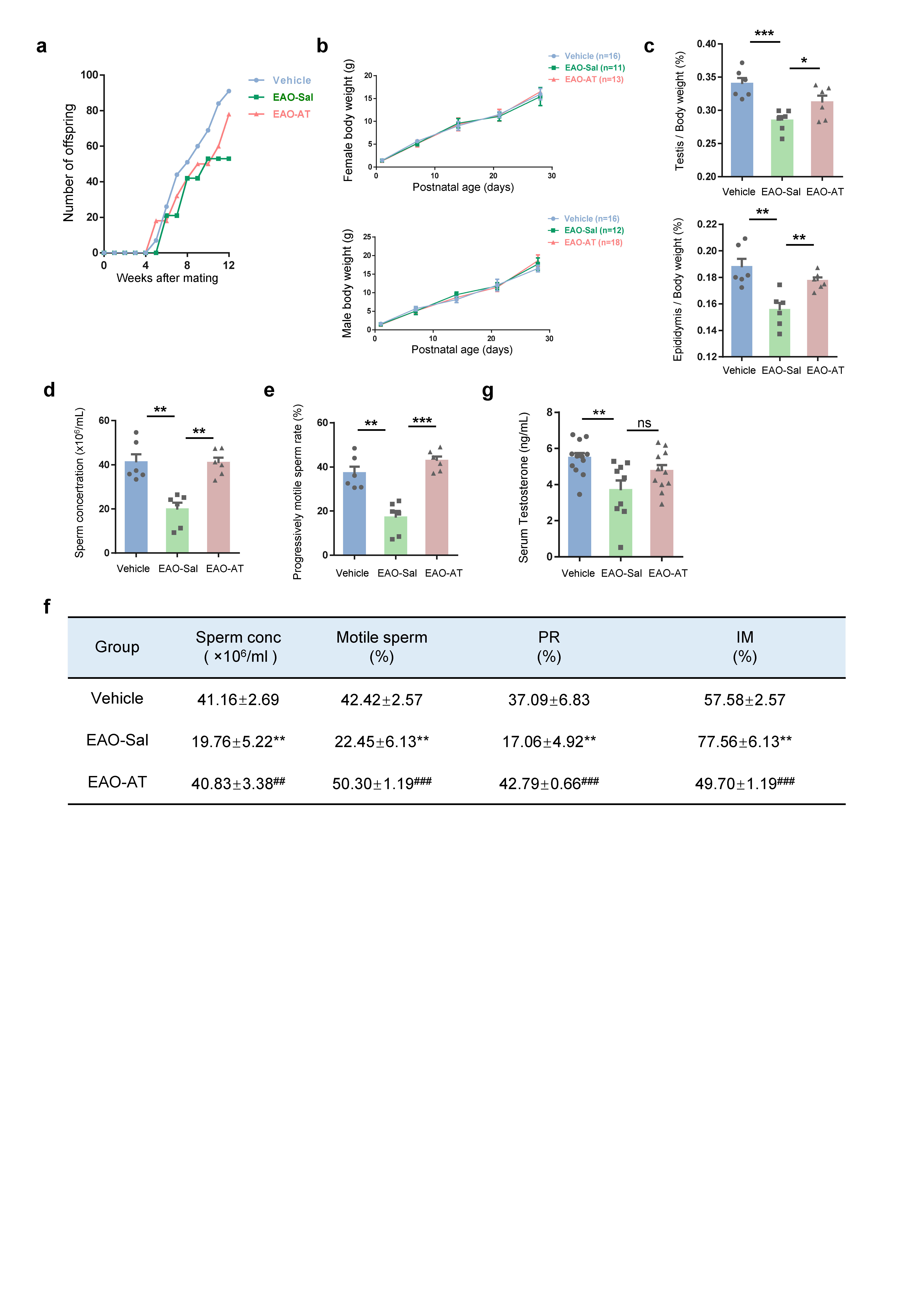
Supplementary Fig. 2: The effect of atorvastatin is not transient.** (a) Number of offspring when natural mating with male mice from Vehicle, EAO-Sal and EAO-AT groups for 10 weeks. (b) Statistical line chart of female (left) and male (right) offspring body weight in the indicated groups. (c) Coefficient of testis (testis/body weight) and epididymis (epididymis/body weight) in Vehicle, EAO-Sal and EAO-AT groups after natural mating, *n* = 6. (d-f) Sperm concentration and sperm motility in Vehicle, EAO-Sal and EAO-AT groups after natural mating, PR means progressively motile sperm rate, IM means immotile sperm rate. *Indicates Vehicle vs EAO-Sal group and # indicates EAO-Sal vs EAO-AT group, *n* = 6. (g) Serum testosterone level of Vehicle, EAO-Sal and EAO-AT groups, *n* = 12. Data are presented as mean ± SEM, * *P <* 0.05, ** *P <* 0.01, *** *P <*0.001, ns indicates no statistical significance.


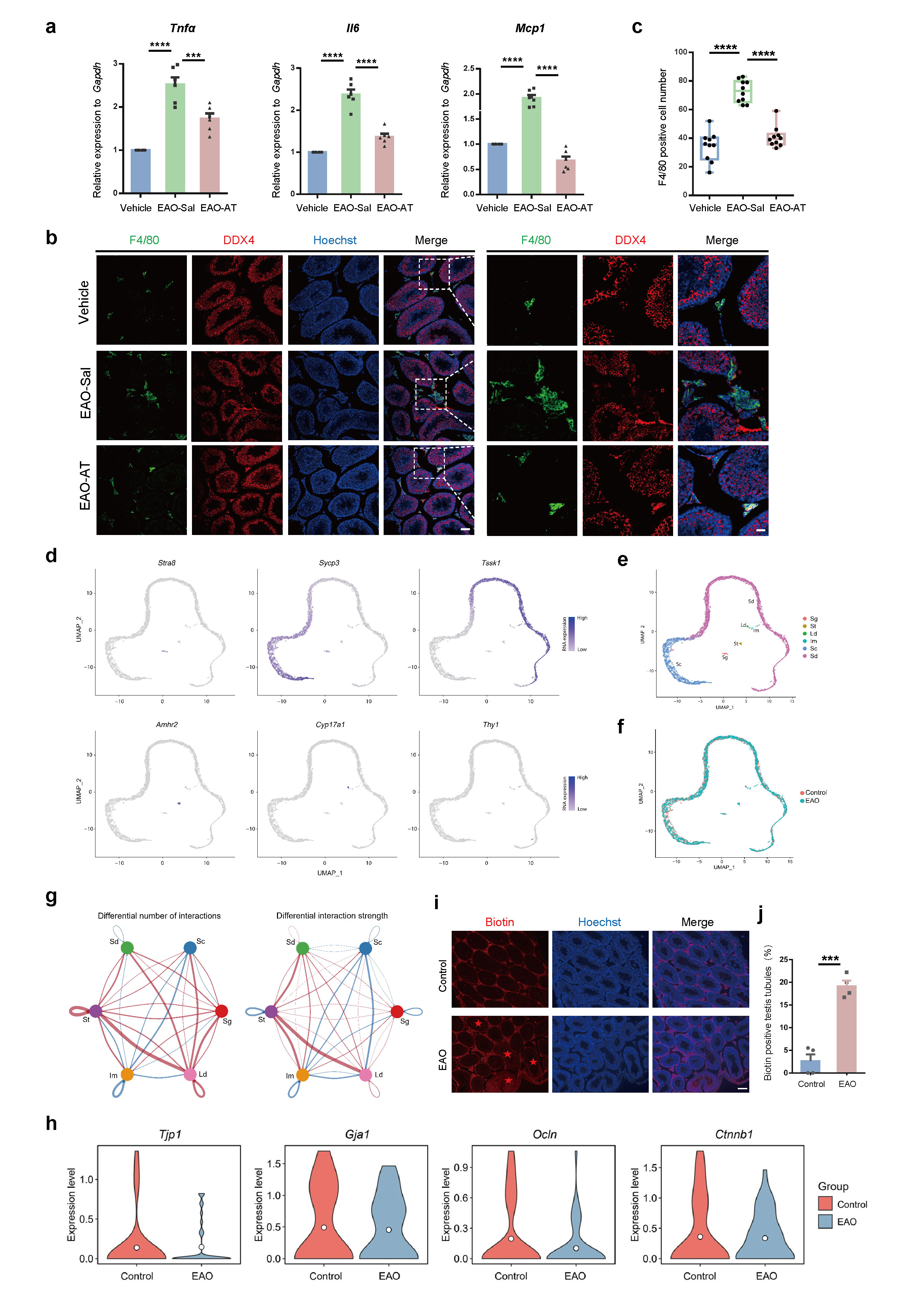


**Supplementary Fig. 3: The analysis of single-cell RNA-seq data indicates notable alterations within the Sertoli cells of EAO mice.** (a) Relative mRNA levels of *Tnfα*, *Il6* and *Mcp1* in the testis from Vehicle, EAO-Sal and EAO-AT groups measured by quantitative PCR. *Gapdh* (Glyceraldehyde-3-phosphate dehydrogenase) was used as internal reference, *n* = 6. (b-c) Immunofluorescence of F4/80 (green) co-stained with DDX4 (red) in testis of Vehicle, EAO-Sal and EAO-AT groups from 10 randomly selected fields. F4/80 labeled macrophages. Scale bar, 50μm. *n* = 4 testis samples per group. (d) Expression of marker genes in different cell types. (e, f) UMAP dimensionality reduction clustering maps of EAO mice (3908 cells) and control group (3021 cells), with cells colored according to cell annotation results (e) or sample source (f). Cell annotations were listed as follows: spermatogonia (Sg), spermatocytes (Sc), spermatids (Sd), Sertoli cells (St), Leydig cells (Lc), and 1 mixture of immune cells (Im). (g) Circle plot illustrates the differences in the number (left) and strength (right) of interactions between EAO mice and the normal control group. The edges connecting the cell types are color-coded, with red indicating increased signaling in the EAO mice compared with the normal control group, and blue indicates the opposite trend. The thickness of the lines represents the number of interactions. (h) Violin plot of corresponding gene expression of BTB related proteins. (i-j) Immunofluorescence of biotin (red) and biotin positive seminiferous tubules percentage in testis of EAO mice and control group. Red asterisks indicate biotin positive seminiferous tubules. Scale bar, 50 μm, *n* = 4. Data are presented as mean ± SEM, *** *P <*0.001, **** *P <*0.0001.

**
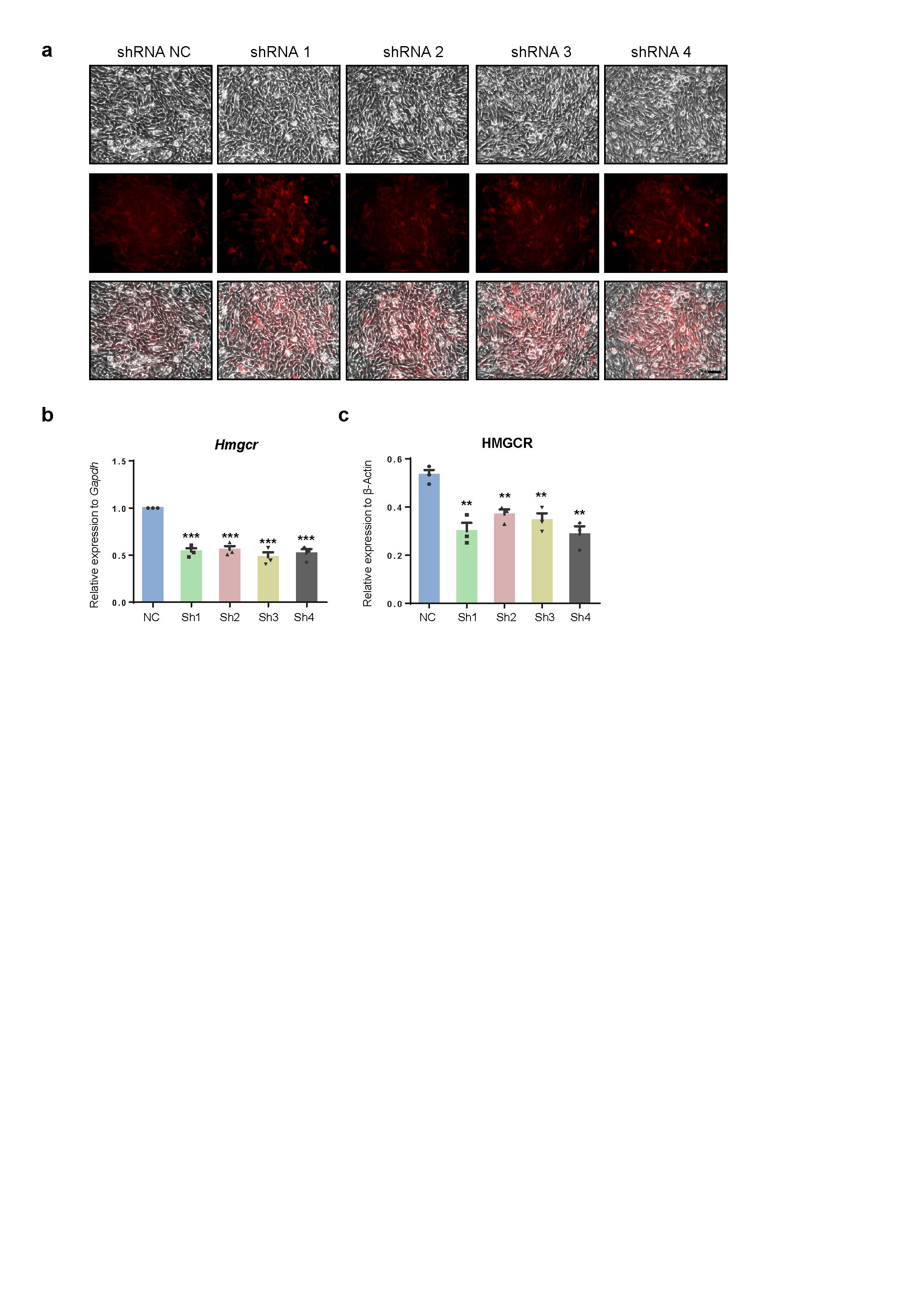
**

**Supplementary Fig. 4: Knockdown of HMGCR in TM4 Sertoli cells.** (a) Representative images showing the TM4-shRNA knockdown cells carrying mCherry red fluorescent labels which helped to assess the efficiency of Lentiviral infection before puromycin selection. Scale bar, 50 μm. (b) Relative mRNA levels of *Hmgcr* in TM4 cells after knockdown with different shRNAs were measured by quantitative PCR. *Gapdh* was used as internal reference, *n* = 3. (c) Grayscale value analysis of western blot for HMGCR in TM4 cells after knockdown with different shRNAs in Fig. 4D. *n* = 3. Data are presented as mean ± SEM, ** *P <* 0.01, *** *P <*0.001.

**
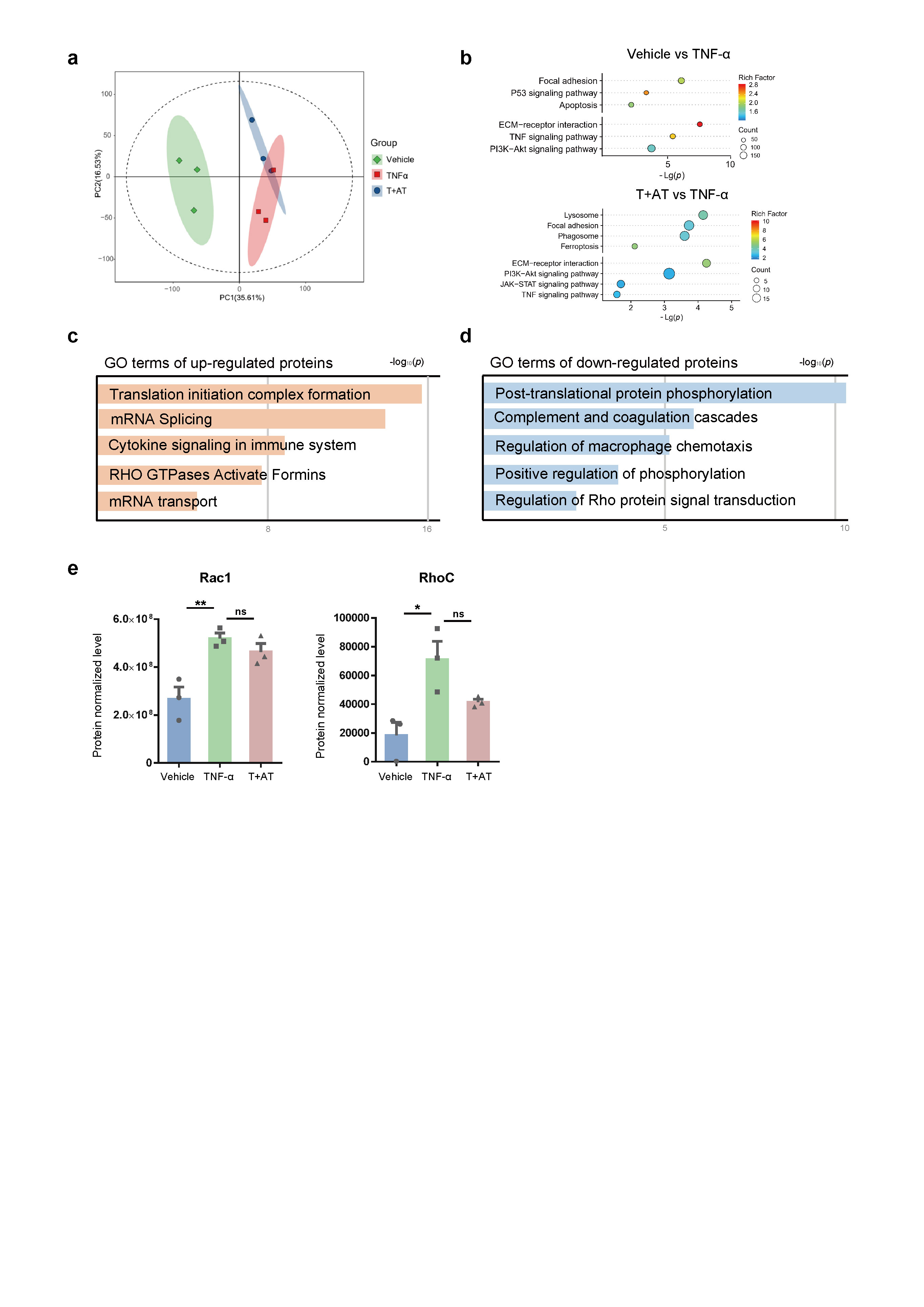
Supplementary Fig. 5: Atorvastatin works by regulating the Rac1/AP1/MMPs pathway in Sertoli cells.** (a) Principal component analysis (PCA) of proteomic data. (b) Major enrichment pathways of KEGG in Vehicle and T+AT groups compared with TNF-α group, respectively. (c) Enriched GO terms of the upregulated proteins in TNF-α group compared with Vehicle group. (d) Enriched GO terms of the downregulated proteins in T+AT group compared with TNF-α group. (e) Proteomics quantitative results of Rac1 and RhoC. Data are presented as mean ± SEM, * *P <*0.05, ***P <*0.01, ns indicates no statistical significance.

**
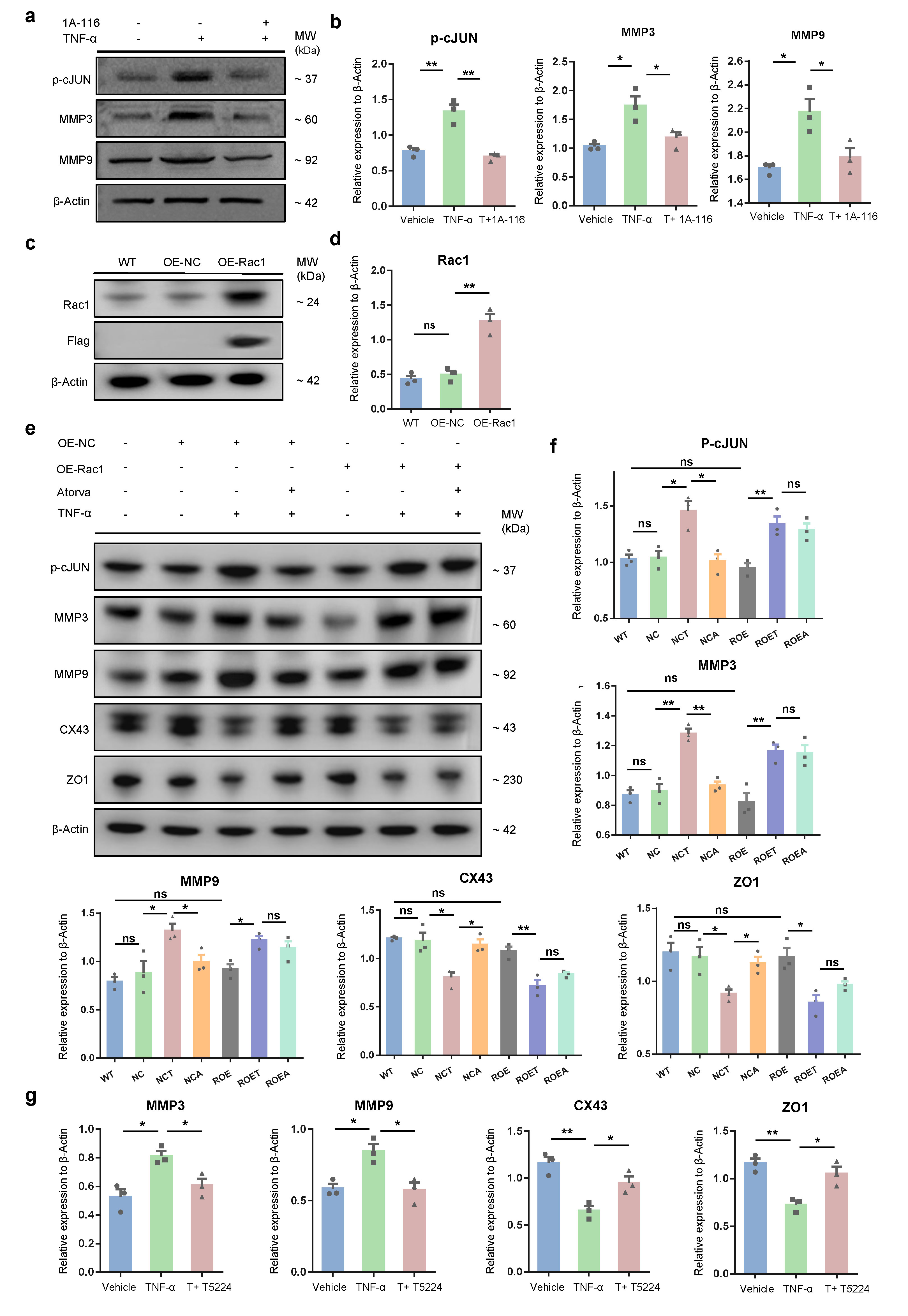
**

**Supplementary Fig. 6: Overexpression of Rac1 counteracted the effects of atorvastatin.** (a) Western blot images of Phospho-cJUN (p-cJUN), MMP3 and MMP9 in TM4 Sertoli cells after 1A-116 treatment. (b) Grayscale value analysis of western blot for Phospho-cJUN (p-cJUN), MMP3 and MMP9 in TM4 Sertoli cells from Vehicle, TNF-α and T+1A-116 groups, *n* = 3. (c-d) Representative western blot images (c) and grayscale quantitative analysis (d) of Rac1 expression in TM4 Sertoli cells: wild-type (WT); negative control lentivirus-infected (OE-NC); and Rac1-overexpressing (OE-Rac1). *n* = 3, β-Actin was used as the loading control. (e-f) Representative western blot images (e) and grayscale quantitative analysis (f) of p-cJUN, MMP3, MMP9, CX43 and ZO1 expression in: wild-type (WT);  negative control overexpression cells (NC) without treatment, with 50 ng/mL TNF-α for 48 h (NCT) or NCT followed by 1 μM atorvastatin for 24 h (NCA); and Rac1 overexpression cells (ROE group) without treatment, with 50 ng/mL TNF-α for 48 h (ROET), or ROET followed by 1 μM atorvastatin for 24 h (ROEA). *n* = 3, β-Actin was used as the loading control. (g) Grayscale quantitative analysis of MMP3, MMP9, CX43 and ZO1 in TM4 Sertoli cells after T-5224 treatment of Fig. 5K, *n* = 3. Data are presented as mean ± SEM, * *P <*0.05, ***P <*0.01, ns indicates no statistical significance.

**
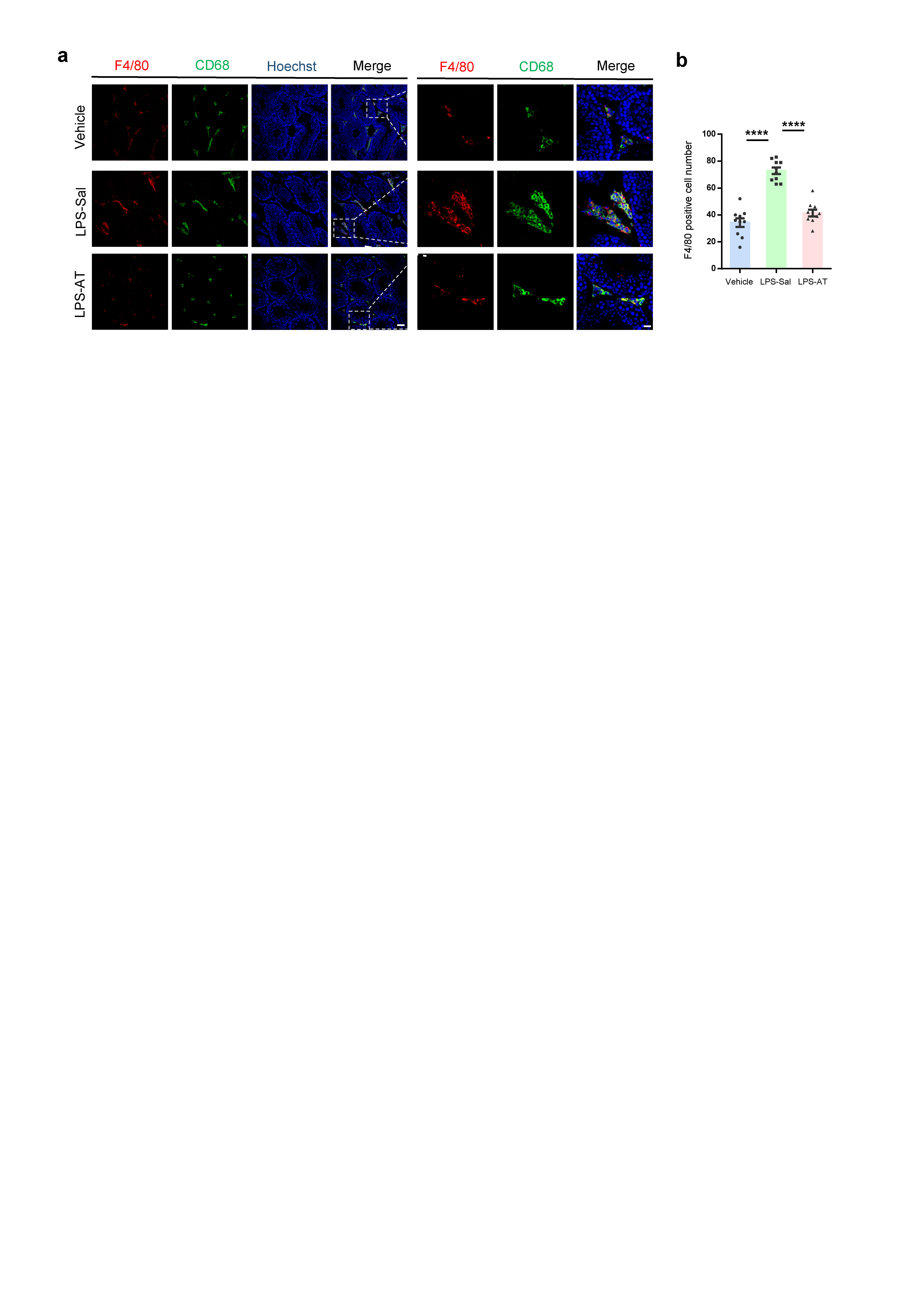
**

**Supplementary Fig. 7: Atorvastatin reduces macrophage infiltration in the testis of LPS-induced chronic orchitis mouse models.** (a-b) Immunofluorescence (a) and analysis (b) of F4/80 (red) co-stained with CD68 (green) in testis of Vehicle, LPS-Sal and LPS-AT groups from 10 randomly selected fields. F4/80 and CD68 were co-labeled as marker of macrophages. Scale bar, 50μm. *n* = 4 testis samples per group. Data are presented as mean ± SEM, **** *P <* 0.0001.
