# Supplementary material for: Atorvastatin improves spermatogenesis in murine and in vitro human chronic orchitis models through restoring blood-testis barriers
Source: Cell Death Discov. 2025 Nov 6;11:505. doi: 10.1038/s41420-025-02749-6 (PMC12592372; doi:10.1038/s41420-025-02749-6)

Full length original western blots

Figure 4a

β-Actin 42 kDa

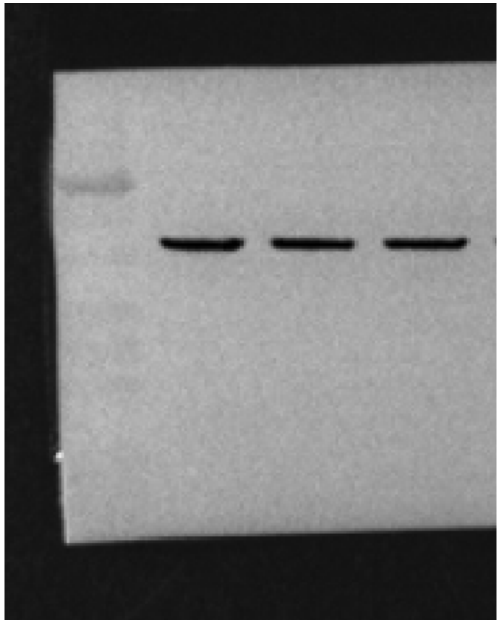

CX43 43 kDa

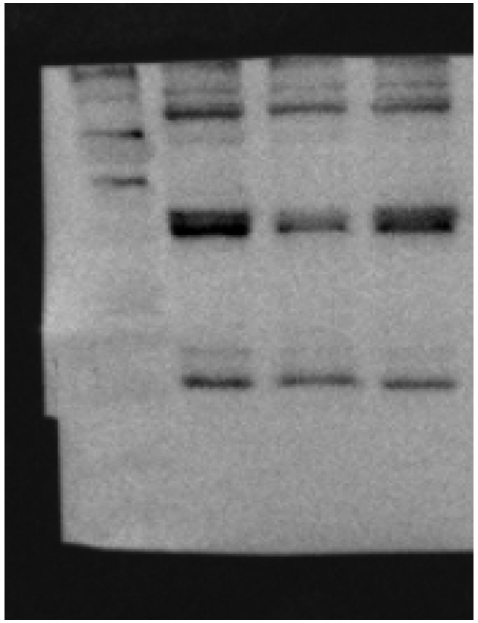

ZO1 230 kDa

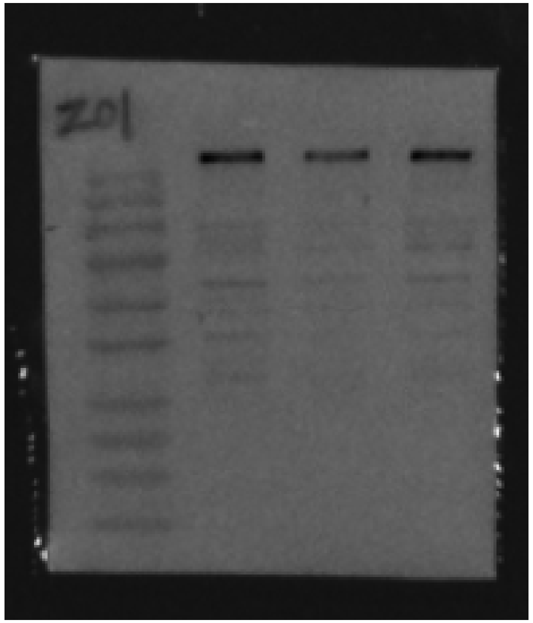

CTNNB1 92 kDa

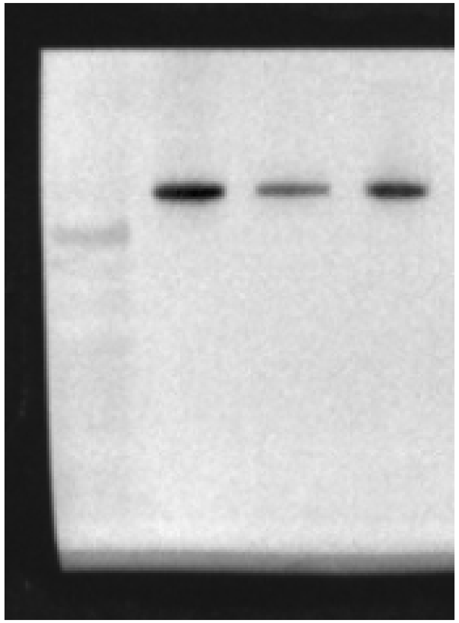

Figure 4d

β-Actin 42 kDa

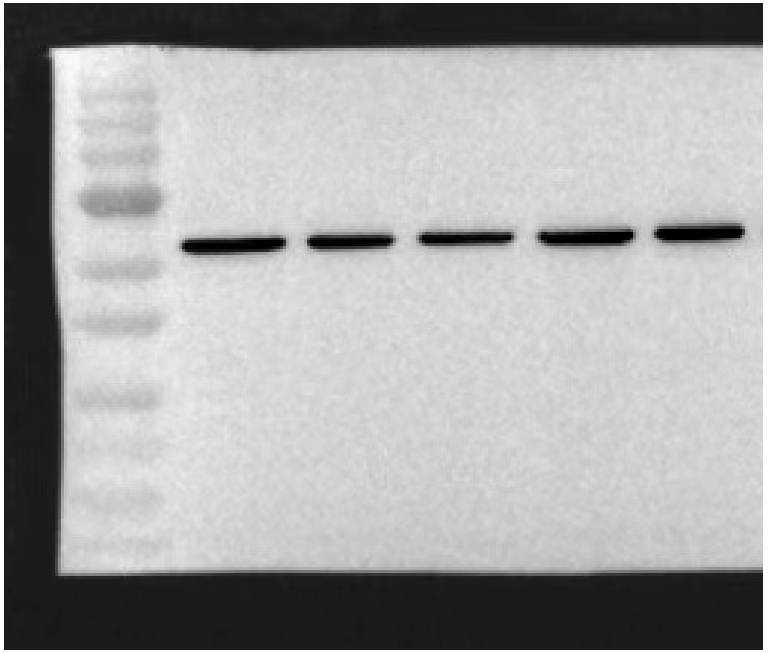

HMGCR 97 kDa

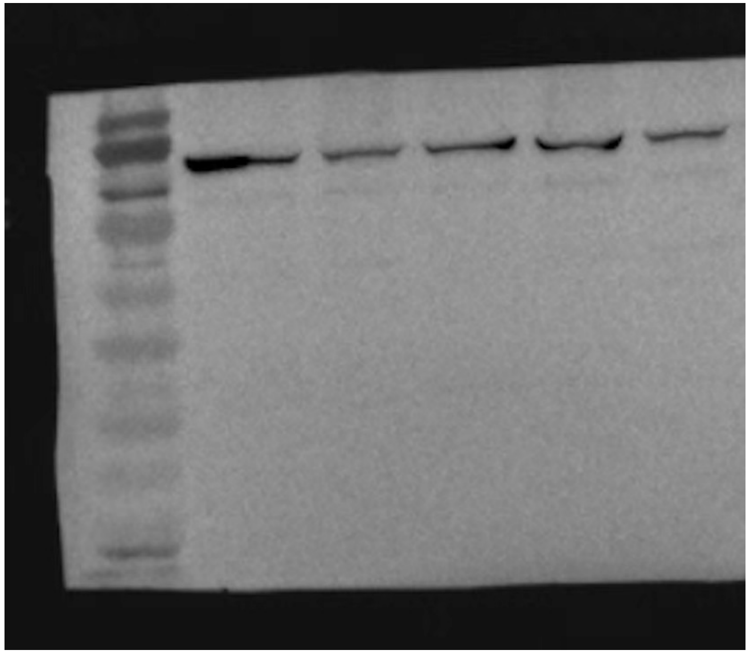

Figure 4e

β-Actin 42 kDa

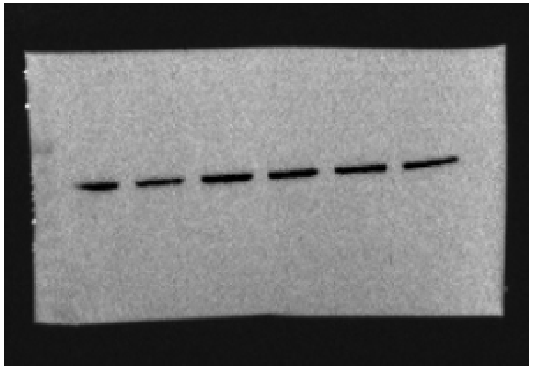

CX43 43 kDa

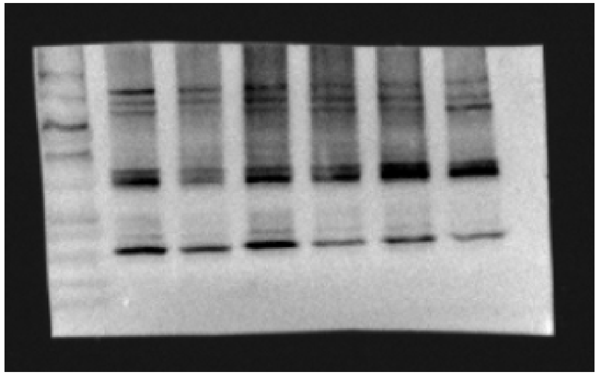

ZO1 230 kDa

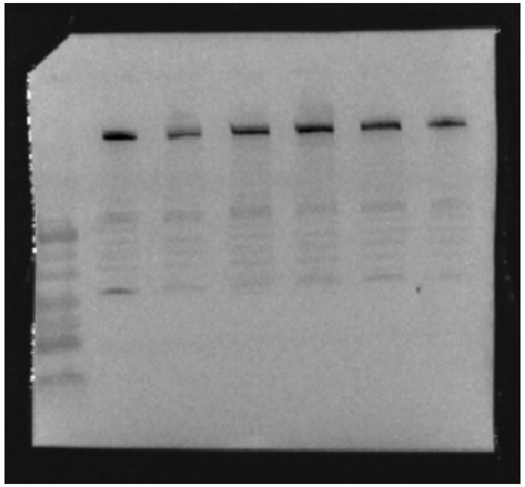

CTNNB1 92 kDa

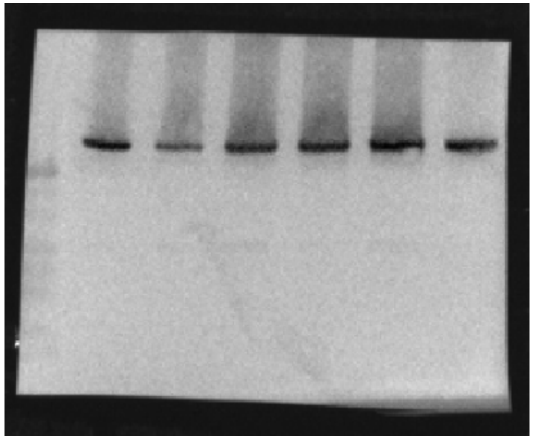

Figure 4g

β-Actin 42 kDa

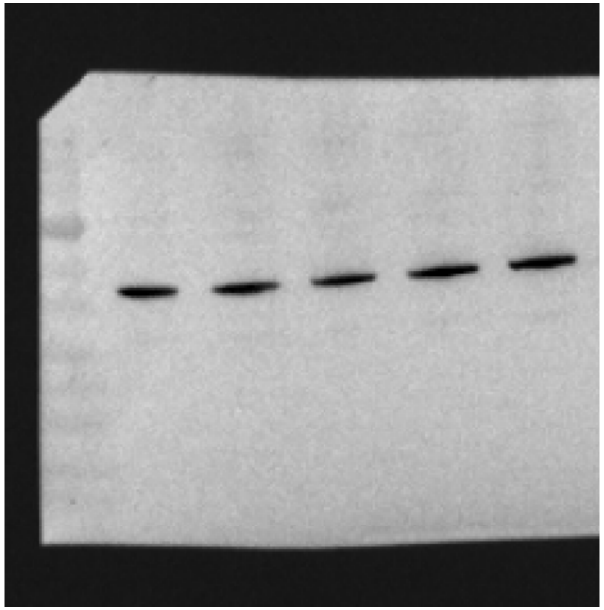

CX43 43 kDa

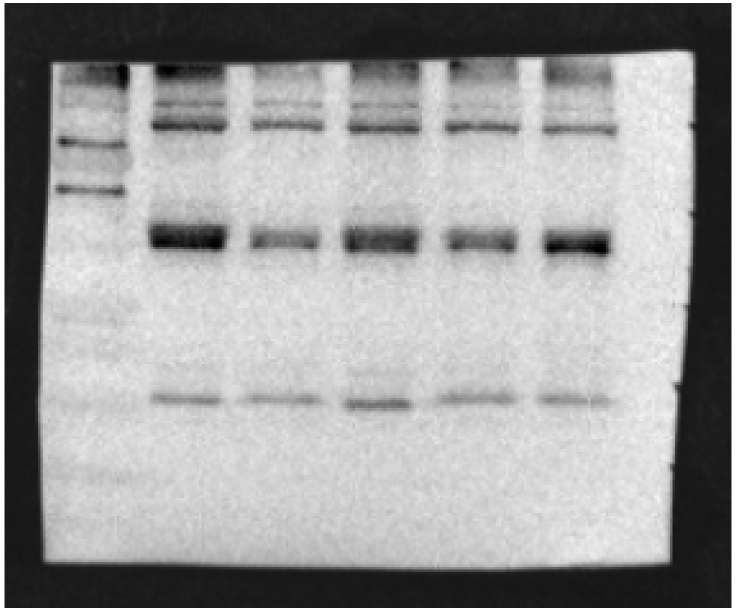

ZO1 230 kDa

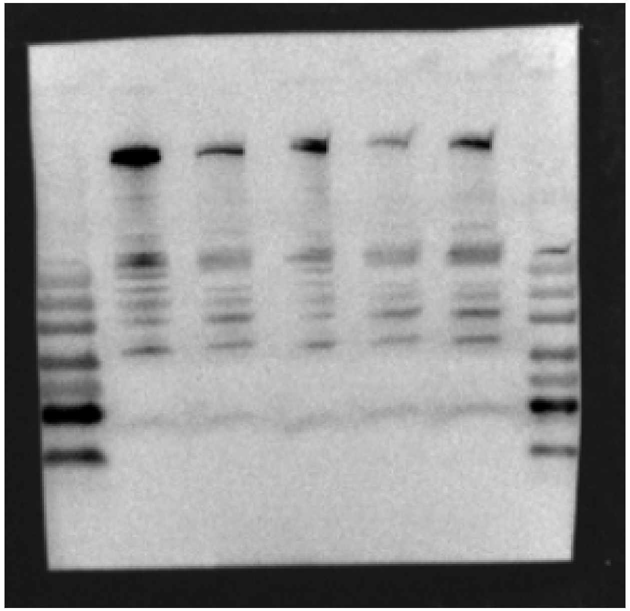

Figure 5d

β-Actin 42 kDa

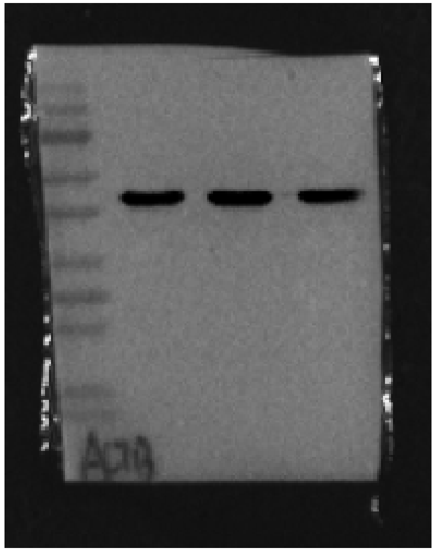

MMP3 60 kDa

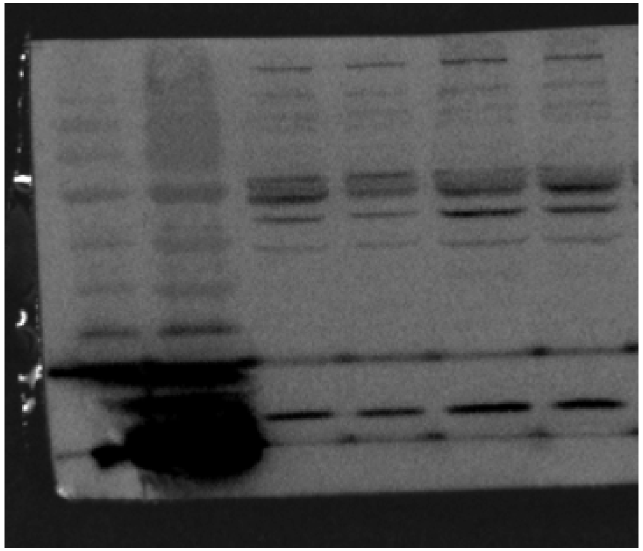

MMP9 92 kDa

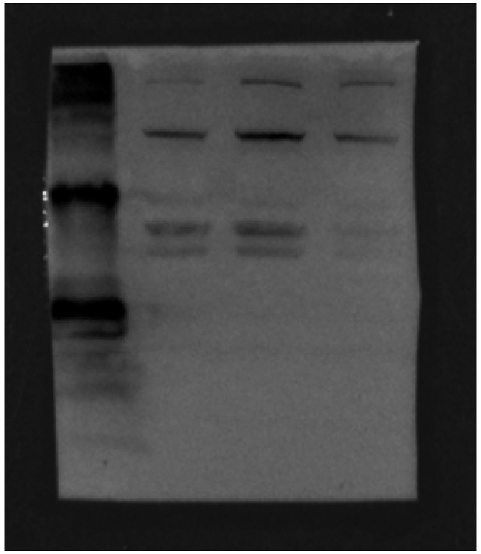

Figure 5h

β-Actin 42 kDa

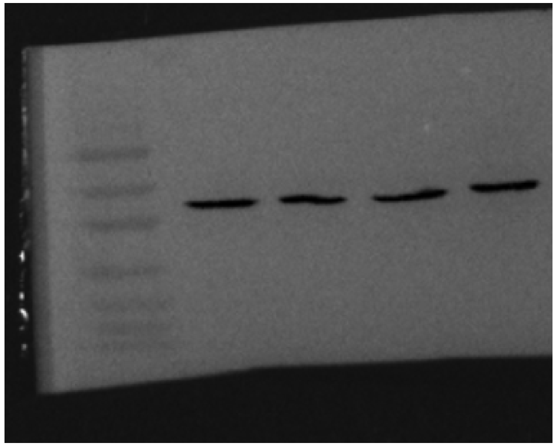

Rac1 21 kDa

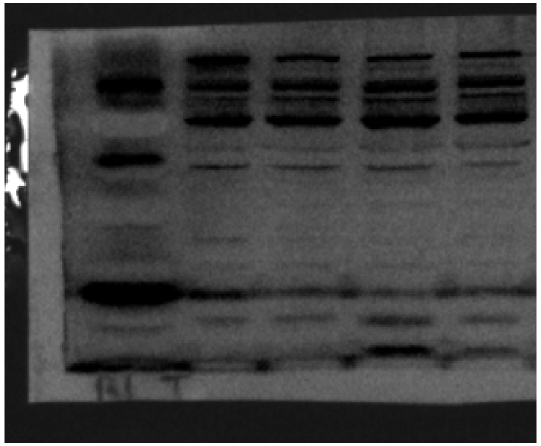

p-cJUN 37 kDa

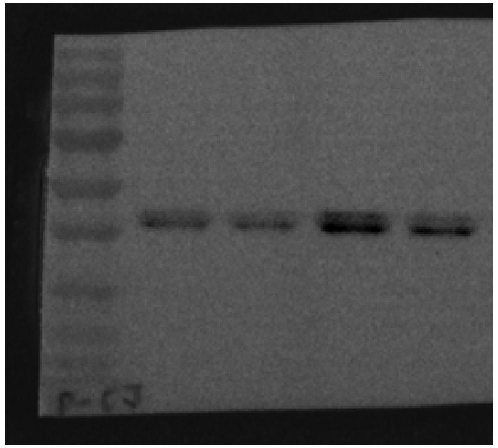

cJUN 36 kDa

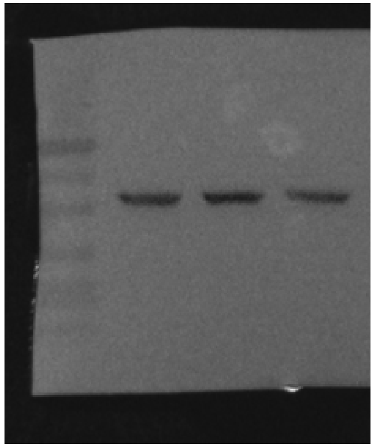

Figure 5k

β-Actin 42 kDa MMP3 60 kDa MMP9 92 kDa CX43 43 kDa ZO1 230 kDa

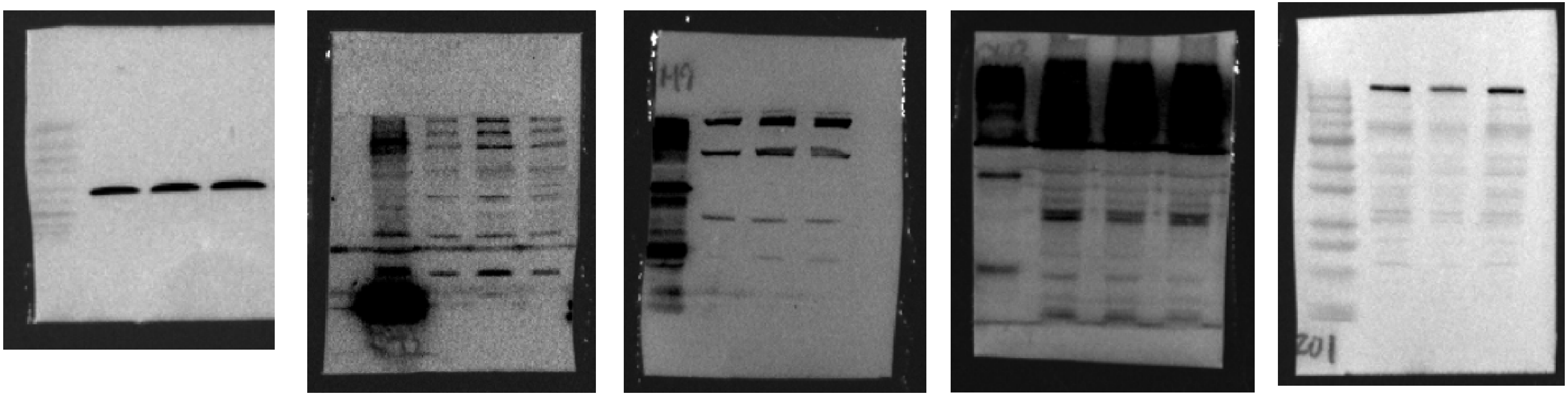

Figure S6a

β-Actin 42 kDa MMP3 60 kDa MMP9 92 kDa p-cJUN 37 kDa

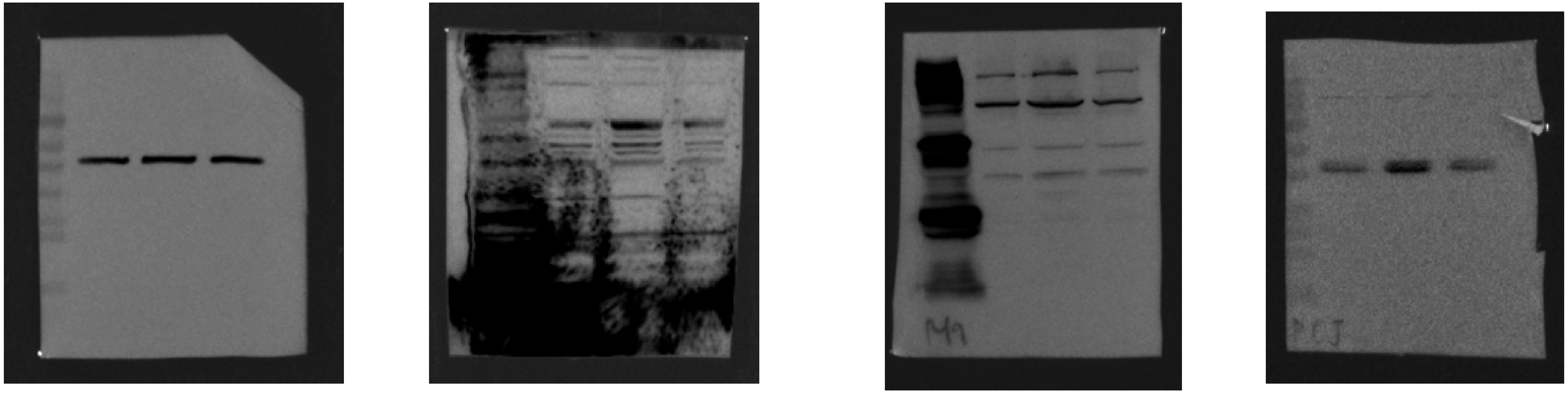

Figure S6c

β-Actin 42 kDa

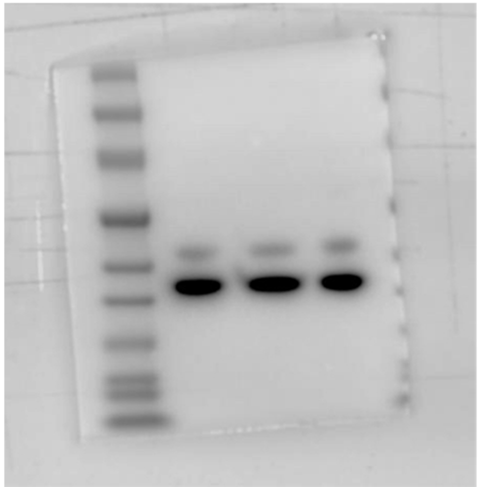

Rac1 (+3X Flag) 24 kDa

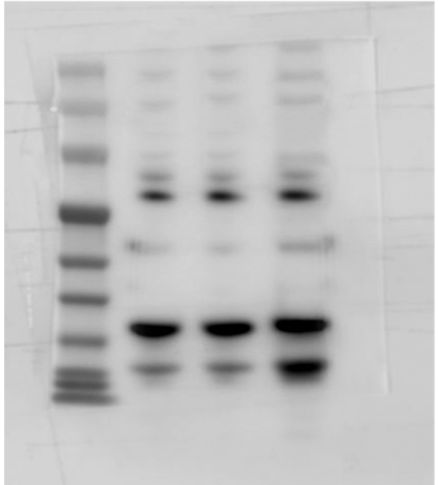

Flag

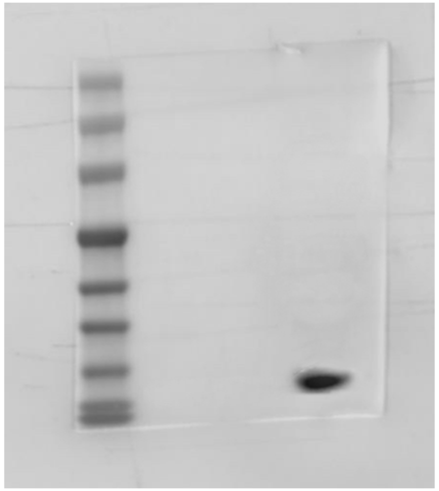

Figure S6e

β-Actin 42 kDa

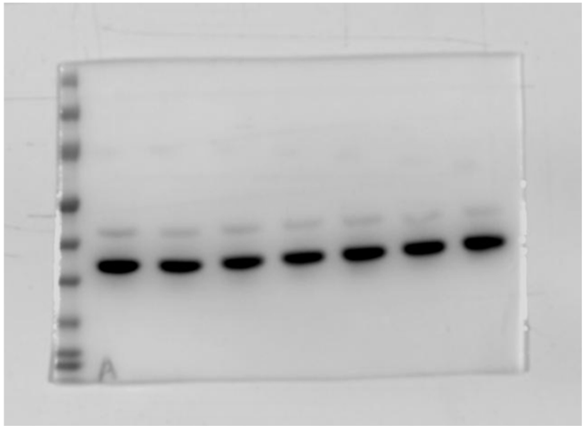

p-cJUN 37 kDa

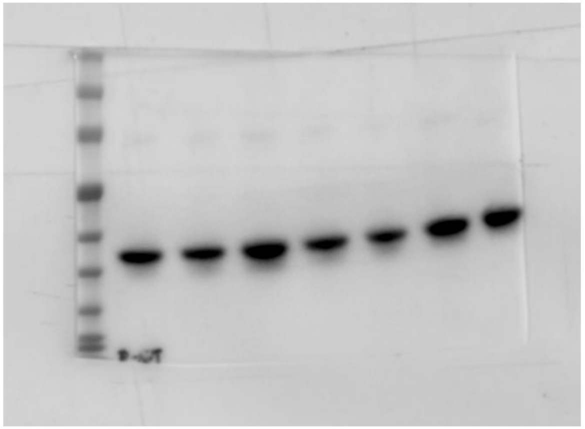

MMP3 60 kDa

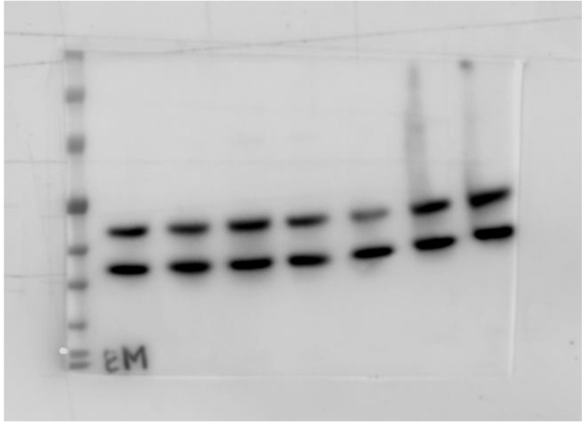

MMP9 92 kDa

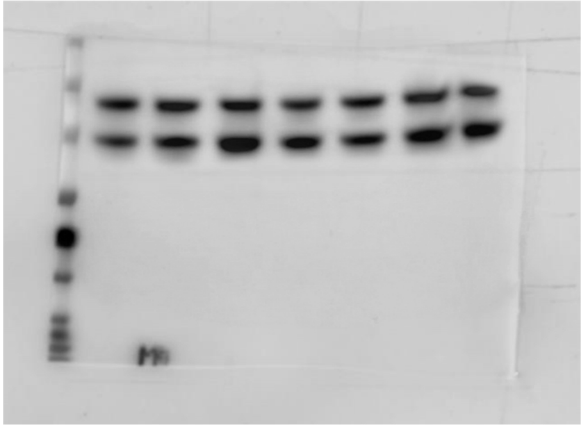

CX43 43 kDa

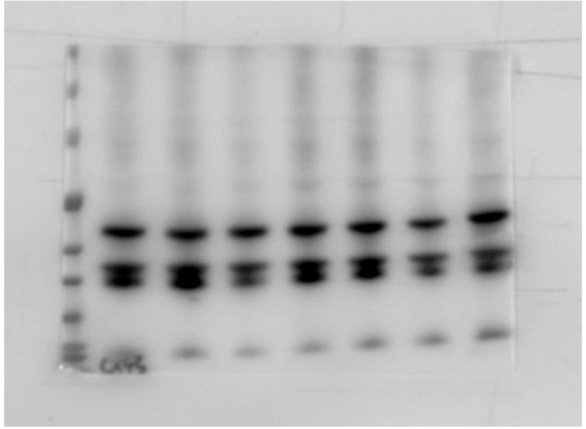

ZO1 230 kDa

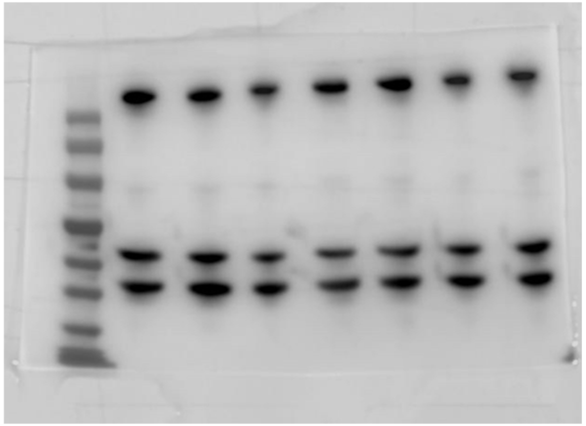

Supplement: Supplementary file 2 — Full and uncropped original Western blots [file 41420_2025_2749_MOESM2_ESM.pdf]
